# Existing evidence on the impact of changes in marine ecosystem structure and functioning on ecosystem service delivery: a systematic map

**DOI:** 10.1186/s13750-023-00306-1

**Published:** 2023-07-20

**Authors:** Carole Sylvie Campagne, Laurie-Anne Roy, Joseph Langridge, Joachim Claudet, Rémi Mongruel, Damien Beillouin, Éric Thiébaut

**Affiliations:** 1 Sorbonne Université, CNRS, Station Biologique de Roscoff, UMR7144, Adaptation et Diversité en Milieu Marin, Place Georges Teissier, 29680 Roscoff, France; 2 Fondation pour la Recherche sur la Biodiversité, Centre de Synthèse et d’Analyse sur la Biodiversité (FRB-Cesab), 5 rue de l’école de Médecine, 34000 Montpellier, France; 3 National Center for Scientific Research, PSL Université Paris, CRIOBE, CNRS-EPHE-UPVD, Maison de l’Océan, 195 rue Saint-Jacques, 75005 Paris, France; 4 Ifremer, University of Brest, CNRS, UMR 6308, AMURE, Unité d’Economie Maritime, IUEM, 29280 Plouzané, France; 5 CIRAD, UPR Hortsys, 97285 Le Lamentin, Martinique France; 6 HortSys, University of Montpellier, CIRAD, Montpellier, France

**Keywords:** Coastal habitats, Biodiversity, Nature’s contribution to people, Spatio-temporal dynamics, Human impacts, Management

## Abstract

**Background:**

The current biodiversity crisis underscores the urgent need for sustainable management of the human uses of nature. In the context of sustainability management, adopting the ecosystem service (ES) concept, i.e., the benefits humans obtain from nature, can support decisions aimed at benefiting both nature and people. However, marine ecosystems in particular endure numerous direct drivers of change (i.e., habitat loss and degradation, overexploitation, pollution, climate change, and introduction of non-indigenous species) all of which threaten ecosystem structure, functioning, and the provision of ES. Marine ecosystems have received less attention than terrestrial ecosystems in ES literature, and knowledge on marine ES is hindered by the highly heterogeneous scientific literature with regard to the different types of marine ecosystem, ES, and their correlates. Here, we constructed a systematic map of the existing literature to highlight knowledge clusters and knowledge gaps on how changes in marine ecosystems influence the provision of marine ES.

**Method:**

We searched for all evidence documenting how changes in structure and functioning of marine ecosystems affect the delivery of ES in academic and grey literature sources. In addition to Scopus, Web of Science, and Google Scholar, we searched 6 online databases from intergovernmental agencies, supranational or national organizations, and NGOs. We screened English-language documents using predefined inclusion criteria on titles, abstracts, and then full texts, without any geographic or temporal limitations. All qualifying literature was coded and metadata were extracted. No formal validity appraisal was undertaken. We identified knowledge clusters and gaps in terms of which ecosystem types, biodiversity components, or ES types have been studied and how these categories are linked.

**Review findings:**

Our searches identified 41 884 articles published since 1968 of which 12 140 were duplicates; 25 747 articles were excluded at the title-screening stage, then 2774 at the abstract stage. After full-text screening, a total of 653 articles—having met the eligibility criteria—were included in the final database, spanning from 1977 to July 2021. The number of studies was unevenly distributed across geographic boundaries, ecosystem types, ES, and types of pressure.

The most studied ecosystems were pelagic ecosystems on continental shelves and intertidal ecosystems, and deep-sea habitats and ice-associated ecosystems were the least studied. Food provision was the major focus of ES articles across all types of marine ecosystem (67%), followed by climate regulation (28%), and recreation (14%). Biophysical values were assessed in 91% of the analysed articles, 30% assessed economic values, but only 3% assessed socio-cultural values. Regarding the type of impact on ecosystems, management effects were the most studied, followed by overexploitation and climate change (with increase in seawater temperature being the most commonly assessed climate change pressure). Lastly, the introduction of non-indigenous species and deoxygenation were the least studied.

**Conclusions:**

This systematic map provides, in addition to a database, knowledge gaps and clusters on how marine ecosystem changes impact ES provision. The current lack of knowledge is a threat to the sustainability of human actions and knowledge-based nature conservation. The knowledge gaps and clusters highlighted here could guide future research and impact the beneficial development of policy and management practices.

**Supplementary Information:**

The online version contains supplementary material available at 10.1186/s13750-023-00306-1.

## Background

In the context of the current biodiversity erosion crisis, there is an increasingly urgent need to manage anthropogenic activities sustainably to conserve and protect nature’s potential to contribute ecosystem services for the benefit of present and future generations [1]. Ecosystem services (ES) and nature’s contribution to people (NCP) concepts have gained interest in their ability to highlight our dependency on nature and all the services we extract from it [2–4]. The concept of ES is relatively recent—being introduced in the late 1970s—and has its roots in the recognition that ecosystems provide irreplaceable goods and services [5, 6]. It has since been largely popularized by the Millennium Ecosystem Assessment as a way of thinking about the relationships between humans and nature [7]. Defined as “the benefits humans obtain from nature” [7], the ES concept helps to produce knowledge to support decisions aimed at promoting nature conservation. The related concept of NCP, defined as “all the contributions, both positive and negative, of living nature to people’s quality of life” [1, 2], popularized first by the Intergovernmental Science-Policy Platform on Biodiversity and Ecosystem Services (IPBES) regional assessments, goes beyond ES by integrating a wider range of specific values and the consideration of negative contributions of nature (also called disservices [8, 9]). Specific values defined by IPBES consider the “judgements regarding the importance of nature in particular situations” and differentiate instrumental, intrinsic, and relational values [10].

These concepts allow for studying socio-ecological systems, which require rigorous approaches across different scientific disciplines—ecology (e.g., [11, 12]), economics (e.g., [13]), anthropology (e.g., [14, 15]), politics (e.g., [16, 17]), or geography (e.g., [18])—to analyse and describe the numerous interactions between living components (i.e., humans and non-humans). The ES concept can improve interactions between disciplines and also among scientists, managers, stakeholders, and politicians by redefining the existing debates on the conflicts between development and conservation [19]. The different ES can be divided into three main categories: (1) provisioning services, which are products obtained from ecosystems (e.g., foods, raw materials for industry); (2) regulation and maintenance services, which are benefits obtained from ecosystems (e.g., climate regulation, coastal protection); and (3) cultural services, which are non-material benefits obtained from ecosystems (e.g., recreative activities) [20–23].

Marine ecosystems provide a wide range of ES. Several lists are available in the literature such as Bordt and Sander [24], Kermagoret et al*.* [25], Barbier [26], and Mongruel et al*.* [15], generally inspired by the classification proposed in Liquete et al*.* [22] and Beaumont et al*.* [27]. For instance, based on the Common International Classification for Ecosystem Services (CICES) [28] and Liquete et al*.* [22], the French platform for the evaluation of ecosystems and ecosystem services listed the ES provided by marine ecosystems [15] as follows: food provision; raw materials from aquaculture; macroalgae production; molecule production; coastal protection; climate regulation; nutrient regulation; pest and disease control; symbolic, emblematic, and aesthetic values; recreation and tourism; landscape amenity; and knowledge production. This study also considered “nursery function” and “maintenance of food webs” in its assessment, even if these are sometimes considered as functions [15]or as regulating services [22]. Although we also included “nursery function” and “maintenance of food webs”, ecological functions, such as primary and secondary production provided by marine ecosystems and sometimes defined as support services, were not included in this review [25, 29, 30].

Marine ecosystems endure numerous direct drivers of change, mainly habitat loss and degradation, overexploitation, pollution, climate change, and introduction of non-indigenous species, all of which threaten the future sustainability of marine and coastal areas [31]. Climate change affects marine ecosystems with different impacts on ES through changes in sea surface temperature, acidification, more extreme events, or sea level rise [32]. The magnitude of the direct drivers may also depend on indirect drivers such as demographic pressure, sociocultural context, economy, technological development, institutions and governance, and conflicts and epidemics. In 2008, a multi-driver analysis showed that no area of the global ocean is unaffected by human influence and that more than 40% of the ocean, mainly in coastal areas (e.g., NE USA, NW Europe, East Asia, Eastern Caribbean) are strongly affected [32]. From 2008 to 2013, “66% of the ocean experienced increases in cumulative human impact […], especially in tropical, subtropical and coastal regions, while only 13% experienced decreases in response to management measures” [33]. Indeed, threats and pressures sustained in marine ecosystems induce changes that have affected the delivery of marine ES, and negatively impacted human health and well-being, especially indigenous peoples and local communities who depend on fisheries [31]. For example, Selim et al*.* [34] highlighted pathways linking fishing and climate (drivers) to spawning stock biomass and recruitment of three demersal fish species (ecosystem processes) and the consequences for delivery of these fisheries and ultimately on food provision (ecosystem services).

In response to growing anthropogenic pressures, marine ecosystems are increasingly included in national and international agendas to counteract the negative impacts of human activities and promote the sustainable use of marine ecosystems (see, for instance, the targets of the Convention on the Biological Diversity or the Sustainable Development Goals of the 2030 Agenda for Sustainable Development adopted by the United Nations). These initiatives are reflected in the implementation of legislation regarding, for example, fisheries management, water quality control, or the establishment of marine protected areas. However, the need to develop effective conservation and protection strategies remains. For instance, marine protected areas involve only about 8% of the marine realm, only partly covering important sites for biodiversity and are not fully ecologically representative, well-connected, or effectively managed [35]. It is therefore crucial to apply rigorous sustainable management practices to help guarantee the delivery of ES and conserve the multiple benefits provided by marine ecosystems that so many people rely on [35, 36]. Hence, it is particularly vital to better understand such ecosystems and highlight the related socio-ecological aspects.

Liquete et al*.* [22] identified, defined, and reviewed the marine ES literature and found 145 articles that specifically assessed marine and coastal ES. That review highlighted that, of the numerous ES provided by marine ecosystems, food provision (i.e., fisheries and offshore aquaculture) seems to be by far the most intensively studied marine ES. Furthermore, it revealed that case studies focused on mangroves and coastal wetlands and were mainly concentrated in Europe and North America. In addition, other specific ecosystems are also frequently spotlighted, such as coral reefs, mudflats, and seagrass beds [15]. Also, knowledge on marine ecosystems seems to decrease with distance from the coastline [15]. Only a few articles have explored ES in deep-sea ecosystems [37]. More recently, systematic maps have been published on the ES provided by the ecosystems in the Baltic Sea (Stories et al*.* [38] and Kuhn et al*.* [39]), revealing cultural services as the most assessed ES category. Likewise, food provision and recreation have been significantly studied in the Baltic Sea, in addition to eutrophication mitigation. The primary focus on food provision stems from the fact that some marine species groups are more assessed and studied, such as commercial species and top predator fish stocks [35]. The ES literature has also been reviewed in IPBES reports and demonstrates, for example, that potential/capacity or the supply component are the central foci in many assessments.

While there are reviews and meta-analyses on marine ES, none deal with the evidence on how ES delivery is affected by changes in marine ecosystems structure and functioning. The need to consider the temporal dynamics in studies is highlighted [40], but the literature seems to focus on snapshot assessments instead on multi-time assessments in relation to ecological dynamics. Thus, our current map was constructed to focus on the literature assessing the impacts of spatio-temporal dynamics of marine ecosystems on the very ES they provide. In addition, we looked at the drivers of change at the origin of marine ecosystems’ dynamics, such as changes in land/sea use, direct exploitation, pollution, climate change, and introduction of non-indigenous species, as well as management effects. We also examined more specifically drivers related to climate change in marine ecosystems with consideration of extreme events (e.g., flood events), sea level rise, warming waters, deoxygenation, or ocean acidification.

The heterogeneity of knowledge in marine and coastal ecosystems and their services is a major obstacle to the effective use of scientific results by decision-makers. A systematic map offers the advantage of structuring existing knowledge to produce results that are useful for decision-making. Following the protocol in Campagne et al. [41], we carried out a systematic evidence mapping exercise to highlight the knowledge clusters and knowledge gaps on how changes in the structure and functioning of marine ecosystems affect the provision of marine ES.

### Stakeholder engagement

Producing this systematic map was part of the InDySEM project [Influence of ecological Dynamics on production and demand for marine Ecosystem Services, funded by the French Foundation for Research on Biodiversity-Centre for Biodiversity Synthesis and Analysis (FRB-CESAB)] and was overseen by both a scientific and a methods team. The scientific team was composed of researchers with expertise on marine ecology, economy, and sociology. The scientific team developed and built the project and advised the project leader and the project officer during regular meetings, who validated any adjustments made to the research topic, the PECO elements (Population, Exposure, Comparator, Outcomes), the search strings as well as all the ROSES elements (see below). The methods team was composed of systematic review and data analysis experts, who followed all the Collaboration for Environmental Evidence (CEE) methodological steps for systematic maps. The FRB-CESAB is a research organization with an international scope whose objective is to implement innovative work on the synthesis and analysis of existing data sets in the field of biodiversity.

### Objective of the review

The main goal of this review was to map existing evidence concerning our primary question: what are the impacts of changes in ecosystem structure and functioning on the services that ecosystems provide?

In addition, the systematic map summarized the evidence database in terms of the following secondary questions:

- What is the existing evidence on how changes in spatio-temporal dynamics of marine biodiversity affect ecosystem disservices?
- What is the existing evidence on how marine ecosystem services and disservices are linked to natural or anthropogenic drivers of change?

Thus, to highlight knowledge gaps on how changes in marine ecosystems affect marine ES, we structured a systematic map according to specific PECO components (Table 1). We focused on changes in biodiversity from the species to the ecosystem level, including functional and structural diversity, and how these changes influence the services provided (i.e., provisioning, regulation, and cultural services). The associated disservices—negative benefits of nature as perceived by humans—were also considered when studied. We focused our systematic map on studies presenting new results of ES change, thus on articles with quantitative or qualitative data, and excluded narrative analyses or articles (e.g., policy reports or reviews without new ES values).

**Table 1:** Components of the systematic map used in this study

PECO element		Definition
Population	Marine biodiversity (ecosystems and species)	Includes all types of marine ecosystems and the species that they contain
Exposure	Changes in marine biodiversity	All changes at all levels, from species to ecosystem, functional and structural
Comparator	Spatial difference—temporal difference	Articles with data at different places (spatial difference) or data on different times (temporal difference)
Outcomes	Marine ecosystem services (and disservices)	All qualitative or quantitative values of marine ecosystem services and disservices

## Methods

The construction of this systematic map followed the methodological guidelines in accordance with the CEE Guidelines and Standards for Evidence Synthesis [42] and conforms to the RepOrting standards for Systematic Evidence Syntheses (ROSES) for Systematic Map Reports presented by Haddaway et al*.* [43] (See Additional file 1). We followed the same methodological protocol as that presented in Campagne et al*.* [41].

### Deviations from the protocol

The protocol [42] was followed. Nevertheless, when we tested the coding strategy (see “Data coding strategy” section), the protocol classification and categories showed some limitations. They were thus more precisely defined or adapted if necessary, according to the coding test process. We refined some categories of metadata and added some new information (i.e., columns) in the evidence base and coded all the information presented in Table 2 (see Additional file 6).

**Table 2:** Metadata (adapted from the protocol in Campagne et al. [41]; *ES* ecosystem services)

Item	Description	References	Adaptation from the protocol
Population			
Ecosystem type^a^	Intertidal rock and other hard substrates; Intertidal sediment; Subtidal rock and other hard substrates; Subtidal sediment; Deep-sea habitats; Pelagic habitats—continental shelf; Pelagic habitats—open sea; Pelagic habitats—estuarine waters; Ice-associated marine habitats + *free space for other ecosystem types*	Classification EUNIS Niveau 2—European Commission	“Circalittoral rock and other hard substrates” were integrated in “Subtidal rock and other hard substrates” because it was difficult if not impossible to differentiate these two categories in most articles
Specific ecosystem^a^	Tidal marsh; Seagrass; Coral reefs; Mangroves; Kelp forests; Beach—dune strip; Estuary + *free space for others specific ecosystem types*	Mongruel et al*.* [15]	Addition of “Beach—dune strip”; “Estuary”
Level of biological organization^a^	The level of biological organization considered in the study: - Species: Species populations (distributions and abundances) or species traits (morphology, physiology, phenology, movement, reproduction); - Community: community composition (community abundance, taxonomic/phylogenetic diversity, trait diversity, interaction diversity); - Ecosystem: functioning and structure.	Pereira et al*.* [58]	We did not code “population”, but “ecosystem” instead because it was more relevant for our map
Characteristics of biodiversity	If any characteristics of biodiversity were assessed in the study, we reported the type of Biodiversity indicator following the definition proposed by Lausch et al*.* [44]: - Taxonomic: the number of different biotic entities (e.g., individuals, populations, species, communities, ecosystems, landscapes); - Structural: the arrangement and distribution (composition and configuration) of biotic entities (e.g., population structure, community structure and landscape patterns); - Functional: the diversity of functions and processes (species processes, community processes and landscape processes).	Lausch et al*.* [44]	None
Biodiversity species^b^	*Free space to record the name of species considered in the study, if any*		New category of coded names of species studies focused on
Outcomes			
Number of ES per Categories	Number of ES for the following ES categories: Provisioning services; Regulating services; Cultural services; Disservices		None
ES	Food provision; Raw materials; Genetic materials; Water provision; Water purification; Air quality regulation; Coastal protection; Climate regulation; Weather regulation; Nutrient cycling; Habitat provision; Pest and disease control; Symbolic and aesthetic values; Recreation and tourism; Cognitive effects; Educational opportunities (related ES terms would be considered in each ES type) + *free space for other ES and for the name of all ecosystem services and disservices in the study with the name as in the study*	Préat [20]	None
ES components^a^	The ES values defined in the study represent: - the ES potential/capacity/supply; “the provision of a service by a particular ecosystem, irrespective of its actual use. It can be determined for a specified period of time (such as a year) in the present, past, or future.” ([45] page 154); - the ES use/flow; “the amount of ES that are actually mobilised in a specific area and time.” ([45] page 155); - the ES demand; “the need for specific ES by society, particular stakeholder groups or individuals. It depends on several factors such as culturally-dependent desires and needs, availability of alternatives, or means to fulfil these needs. It also covers preferences for specific attributes of a service and relates to risk awareness”. ([45] page 156) (Indication were added if it is preferences/desires; the ES benefits or another form of demand or when the ES demand is not specified).	Following definitions in Burkhard and Maes [45]	We grouped the ES components differently because definitions vary among authors; we grouped components with closed definitions
ES values	The type of ES values are coded based on the ES indicators in the study following the definitions in IPBES Values Assessment Report 2022: - “Economic values are based on individual preferences, reflecting individual needs, wants, perceptions, and worldviews, as well as the scarcities imposed by nature and by the social and economic contexts within which people live”; - Sociocultural valuation methods aim “to value nature and its contributions to people by discovering the psychological, historical, cultural, social, ecological, and political contexts and conditions, as well as the worldviews and social perceptions that shape individually held or commonly shared values”; - “Biophysical approaches assess value based on the intrinsic properties of objects by measuring underlying physical parameters. They generally aim to examine the ecological importance of attributes, qualities, and quantities characterizing nature’s condition and functioning”.	Following definitions in IPBES Values Assessment Report p. 17 [46]	None
Exposure/comparator			
Scale of study area	Subnational; National; Supranational; Continental; Global; No case study	Liquete et al*.* [22]	“Local” was integrated in “subnational” because it depended on the size of the study in the article and the country involved
Study country	Country included in the study; global		None
Study ocean locality^a^	Ocean included in the study based on the case study		New category
Study sea locality^a^	Sea included in the study based on the case study or NA		New category
Specific location	*Free space for the name of the case study site*		None
Number of sites	Number of case study sites reported in the study		New category
Temporal scale interval raw data^a^	Interval of time elapsed between successive temporal replicates of the raw data (i.e., the data used for the analysis in the article, e.g., data used to calibrate a prediction)		Distinguishes information in terms of raw data (which we defined as the data used for the analysis in the article, e.g., data used to calibrate a prediction) and the results data (i.e., the data results of each study, e.g., the results from a prediction model)
Temporal scale duration raw data^a^	Duration of time elapsed between first and last temporal replicates of the raw data analysis		
Temporal scale interval result data^a^	Interval of time elapsed between successive temporal replicates of the result data (i.e., the data results of each study, e.g., the results from a prediction model)		
Temporal scale duration result data^a^	Duration of time elapsed between first and last temporal replicates of the result data analysis		
*Time frame*^a^	- Past: data prior to 3 years before the date of publication; - Present: data in the last 3 years before publication; - Future: data after the publication.		None
Time data^a^	- Observation and descriptive study with measurement of a specific parameter; - Experimentation and demonstrative study with experiments showing causality effects between factors; - Prediction/projection: definition of potential values in the future based on models. Projection is future when a change/pressure happens. Prediction is future when nothing influences the evolution.	Adapted from Sordello et al. [47]	Addition of “experimentation” and addition of “projection” with “prediction”
Pressure type^a^	Land/sea use change; Direct/overexploitation; Pollution; Introduction of non-indigenous species; Management effects; Climate change (CC); CC– extreme events; CC—sea level rise; CC—warming waters; CC—deoxygenation; CC—ocean acidification; CC—other pressure + *free space for other pressures* (related to climate change, e.g., the impact of El Nino Southern Oscillation)	IPBES [1] and Halpern [32]	We added Climate change pressures adapted from detailed Halpern [32] as it is a specific focus we wanted
Type of management^a^	Marine protected area; Water quality management; Fishery management		We changed this item to specify the type of management or the presence of a marine protected area
Complementary information			
Type of data^a^	- Primary data: data was created and not based on other studies; - Quantitative data: empirical or observational data or biophysical or economic indicators; - Qualitative data: data from interviews or public perceptions from questionnaires; - Data variability: when an indicator of the variability is presented in the article.	Following Langridge et al. [48]	Added categories
Study design^b^	- Control-impact design: two or more ecosystems/areas/species with at least one with the driver of change and at least one without the driver of change, both studied at one point in time; - Before-after design: one ecosystem/area/species studied before and after an event (e.g., a new driver of change or a sudden event as an extreme climate event); - Before-after control-impact design: two ecosystems/areas/species; one with the driver of change and one without, both at two time points: before and after the event; - Multiple before-after control-impact design: two or more ecosystems/areas/species: two or more with the driver of change and several without, all at several time points, before and after the event; - Multiple impact design: two or more ecosystems/areas/species with different characteristics (e.g., exposed to different drivers of change) compared at 1 time point; - Multiple impact design—a temporal series: two or more ecosystems/areas/species with different characteristics over time; - Temporal series during a disturbance: one ecosystem/area/species or several studied over time when exposed to a chronic disturbance; - Temporal series post-disturbance: one ecosystem/area/species or several studied over time post-disturbance; - Correlation analysis: correlations between the magnitude of a driver of change and one or several ecosystems/areas/species characteristics	Adapted from Sordello et al. [47]	New category

^a^Category modified from the protocol Campagne et al.[41]

^b^New category; not in the protocol Campagne et al.[41]

### Search for articles

#### Search string

The search string was composed in accordance with the key components of the question representing Population, Exposure, and Outcomes as planned in the protocol [41] and Table 1. The search string used on the Web of Science in “exact search” mode is presented in Table 3. The asterisk (*) at the end of a search term/word was used to accept any variant of a base term. The dollar sign ($) was used to accept single or no added characters, useful for retrieving plural and singular forms. Quotation marks were used to search the exact word order.

**Table 3:** Search strings and search hits

Name	Search field	Search string	Search hits	Date of search (DD/MM/YYYY)
Literature databases				
Web of science	TS	((marine OR coast* OR ocean OR sea OR littoral OR maritime) AND (species OR biodiversity OR ecosystem OR ecological) AND (“ecosystem service$” OR “contribution to people” OR “ecosystem function$” OR “ecosystem process” OR “landscape service$” OR disservice$ OR “provisioning service$” OR ((provision OR production OR exploitation) AND (food OR fisher* OR macroalgae$ OR molecules)) OR “biomass for nutrition” OR “biomass for materials” OR “genetic materials” OR “raw materials” OR “maintain* food webs” OR “life cycle maintenance and habitat protection” OR “habitat provision” OR “nursery function” OR “regulation service$” OR “climate regulation” OR “carbon sequestration” OR “weather regulation” OR “atmospheric composition and conditions” OR “air quality regulation” OR “coastal protection” OR “water retention” OR “nutrient regulation” OR “nutrient cycling" OR “pathogen regulation” OR “pest and disease control” OR “mediation of waste” OR “mediation of mass” OR “cultural service$” OR “intellectual interaction” OR “physical interaction” OR “experiential interaction$” OR tourism OR recreation OR amenity OR aesthetic OR heritage OR symbolic OR “cognitive effect$” OR “knowledge production” OR education) AND (dynamic$ OR impact$ OR effect$ OR variation$ OR interaction$ OR evolution OR change$))	17329	20/07/2021
Scopus	TITLE-ABS-KEY		24051	20/07/2021
Online search engine				
Google scholar	Keywords	(Marine OR coastal OR ocean) AND (species OR biodiversity OR ecosystem) AND “ecosystem services” AND change	300	22/07/2021
Organizational websites				
FAO	Language: “English”	Fishery	50	27/08/2021
UNESCO	Filter: language: “English”—source: “UNESCO”—AuthoCorporate-en-s: “Intergovernmental Oceanographic Commission”—nature of content: “guide” AND “manuals and handbooks”	Marine ecosystem service	50	19/08/2021
UNEP	Filters: ”Reports and publications” AND “Publication” AND “Report”, “Ecosystems and biodiversity” AND “oceans and seas"	Marine ecosystem service	50	19/08/2021
US NOAA		Ecosystem service	15	19/08/2021
EEA		Marine ecosystem service	7	19/08/2021
IUCN		Ecosystem service	32	27/08/2021

The search terms used for the substring on ES types included different synonyms for each ES in order to be as inclusive as possible, inspired by different lists of marine ES based on Mongruel et al. [15], the Global Ocean Accounts Partnership [21] and Liquete et al*.* [22]. The search terms for the substring on Exposure, which involves changes in biodiversity (from species to ecosystems) were composed of key words synonymous to “change”. The search string was tested and constructed in the Web of Science Core Collection (WOS) to obtain the highest efficiency and the best comprehensiveness related to the test list (see Additional file 2). Searches were performed using English terms only. All relevant international literature published in English was included in this systematic map, including diverse bibliographic documents (e.g., books, journal articles, theses and technical reports).

#### Search sources

Publication databases, on-line search engines, and the organizational websites were searched without any time restriction (e.g., since 1788 for Scopus). All searches were undertaken between July and August 2021 (Table 3).

#### Bibliographic databases

Title, abstract and keywords of the Scopus and WOS publication databases were searched using the search tags “TITLE-ABS-KEY” and “TS”, respectively. All databases were accessed with the subscription of the French National Centre for Scientific Research (CNRS).

#### Search engine

A supplementary search in Google Scholar, with the aid of Publish and Perish [49] software, was used to retrieve additional literature. Google Scholar’s use of Boolean characters differs from WOS and Scopus and is limited in terms of the number of characters, and thus search terms [50]. Therefore, we adapted the search string to correspond to what the review team deemed as the most important keywords and used the “keywords” field to search the title, abstract, and body of text with the following keywords: “(marine OR coastal OR ocean) AND (species OR biodiversity OR ecosystem) AND “ecosystem services” AND change”. We exported the first 300 search hits, in line with the recommendations by Haddaway et al*.* [50].

#### Grey literature searches

Six specialist organization websites were searched (cf. Table 3) to collect technical reports with primary data related to our question. For each organizational website, the use of specific keywords with manual-searches varied between website as presented in the methodological protocol (Campagne et al*.* [41]) and as listed in Table 3*.*

The keywords used were “marine ecosystem services”, which contains the keywords for the Population and the Outcomes components. Adaptation of the keywords used depended on the main topic of the organizational website. For example, because NOAA focuses on marine ecosystems, the search string was only “ecosystem services”. For the FAO, the main keywords did not lead to any results, so we focused on one ecosystem service: “fishery”. Again, the main keywords did not lead to any results in the IUCN publication websites, so we focused only on “ecosystem service”. Other websites were tested such as the Intergovernmental Panel on Climate Change (IPCC) and the IPBES websites. Nevertheless, the main keywords of our search string did not lead to any results. These intergovernmental websites only offered review reports and no records with primary results. A maximum of 50 references was considered for each organizational website.

#### Estimating the comprehensiveness of the search

The search terms were tested in WOS. The review team compiled a list of 30 articles that we considered as important and relevant for our respective fields and the research topic. These articles are listed in Additional file 3. Search terms were modified and refined until these benchmark publications were retrieved. For example, words related to Population, Outcome and Exposure were progressively added as described in Additional file 2. In WOS, 25 out of the 30 articles in our test list were retrieved with the final search terms, with 2 articles were not found due to the search string and 3 out of the 30 articles were not found at all in WOS but only in other literature database. With all the results extracted (WOS, Scopus and Google Scholar), 29 out of the 30 articles in our test list were retrieved, indicating a 96.7% comprehensiveness (Additional file 3). The only article we did not retrieve was Roessig et al*.* [51]. We tried different search strings; nevertheless the numbers of documents found with other search strings retrieving Roessig et al*.* [51] were either unmanageably high or other documents in the test list were not found. The current search string at 96.7% comprehensiveness was assumed to be the best compromise.

#### Assembling and managing search results

Once the extraction of records from each database and website was completed, we reassembled all records from all the different sources into one spreadsheet file. To do so, records from Scopus, WOS, and Google Scholar were re-exported from Zotero and Mendeley to import the same file types into the R environment for correct merging of records from the different sources and formatting of data columns. Records from organizational websites were manually added in the final Excel files.

We removed clear and partial duplicates based on similar DOI and similar titles using R package revtools [52] and the “find_duplicates” function. In addition, we used the “check duplicates” function in Microsoft Excel software for a double verification.

#### Article screening and study eligibility criteria

##### Screening process

A three-stage filtering process was undertaken in accordance with pre-defined screening and study eligibility criteria [41]. Titles were screened first, followed by abstracts, then full texts.

Full texts were sought for all selected abstracts using the journal subscriptions via the CNRS and Sorbonne University. If the articles were not retrievable, requests for full texts were made via ResearchGate (www.researchgate.net), or the authors were contacted directly through ResearchGate or by email. We integrated full texts found or received until 28 February 2022. Unretrievable full texts of accepted abstracts were not screened. Incomplete texts were considered as not found. They are listed in Additional file 4.

We applied a conservative approach: titles or abstracts that did not clearly fit the inclusion criteria or did not clearly fit the exclusion criteria (details below in the Eligibility criteria section) were kept for the next eligibility screening stage. No screened article was authored or co-authored by the screener.

To test the consistency of the screening process, Cohen’s kappa coefficient [53] was calculated on a list of similar articles screened independently by two screeners. But before the statistical tests were run, a training phrase was undertaken. The two screeners met to practice, discuss and adapt the eligibility criteria on 100 test titles and then on the abstracts of these accepted test titles. The goal of these meetings was to verify the understanding of the eligibility criteria.

The kappa tests were then run on 1000 titles out of the 41 884 records (2.38%) (due to resource limitations and the considerable number of records within all databases used, it was not possible to run the kappa test on 10% of the titles). Cohen’s kappa coefficient for the title screening stage was 0.83. At the abstract screening stage, we tested 402 of the 3999 titles (10%) selected and Cohen’s kappa coefficient was 0.70. Finally, on 116 full texts of the 1119 full texts retrieved (10%) Cohen’s kappa coefficient was 0.87. At each screening stage, the reviewers met to discuss all remaining discrepancies.

##### Eligibility criteria

The selection of records depended on the inclusion and exclusion criteria presented in Table 4. The inclusion/exclusion decisions were reported at each screening stage. In line with the guideline recommendations, reasons for exclusion during the full-text screening were also reported (see Additional file 5).

**Table 4:** Eligibility criteria to select articles to include in the systematic map

Criterion	Screening step	Inclusion criteria	Exclusion criteria
Population	Title	Articles whose title deals with biodiversity, i.e., species, habitats, and/or ecosystems in marine environments. Non-exhaustive examples may include open-ocean, continental shelf, coastal areas, seagrass meadows, estuaries, mangroves, coral reefs, etc	Articles whose title explicitly only refers to terrestrial and/or freshwater biodiversity, species, habitats or ecosystems, i.e., articles regarding exclusively aquatic species and habitats (e.g., lakes, floodplains, rivers, subterranean habitats, etc.) or to terrestrial species and habitats (e.g., forest, agricultural ecosystems, etc.)
Outcomes	Title	Articles dealing with marine ecosystem services (as well as related terms such as “nature’s contributions to people”). (e.g., blue carbon sequestration, snorkelling, whale watching) Articles dealing with the marine ecosystem service of food supply in terms of indicators of stock or population size of commercial species (e.g., fishery stock)	Articles dealing solely with function or structure processes and not related to effects on ecosystem services (e.g., primary production, photosynthesis) Studies only addressing species criteria with indicators other than the stock or the population size of the species (e.g., species distribution)
Exposure	Abstract	Any article or study exposing marine biodiversity, i.e., species, habitats, and ecosystems, to a change in structure and/functioning over time caused by an agent of change, i.e., human activity (e.g., direct/overexploitation, land/sea use change, etc.) or a change caused by different spatial areas studied	Articles presenting no exposure to a change
Comparator	Abstract	Articles studying changes in ecosystem services through time or space (i.e., temporal or spatial comparisons). This may mean a different study type as detailed in Table 2. Accepted with synchronic comparators (same time, different sites)	Articles only assessing ecosystem services at one time or in one site/area
Temporal period	Abstract	Articles analysing relevant outcomes with data covering periods of at least part of the twentieth century and/or the twenty-first century	Articles analysing data covering periods ending before 1900 (e.g., palaeoecology analysis)
Outcomes	Full text	Articles analysing relevant outcomes containing qualitative or quantitative values of marine ecosystem services and disservices	Articles without qualitative or quantitative values of marine ecosystem services and disservices (e.g., narrative review, opinion paper, policy paper without new quantitative or qualitative values defined)

Regarding title screening, only articles with a clear mention of “marine ecosystems” and “ecosystem services” with the wording of ES or ES-related concepts directly mentioning an ES were accepted (see list in Liquete et al*.* [22], or Préat [20] for a list of marine ES). In the abstract screening process, in addition to the previous criteria, we considered Exposure and Comparator. If an article fit the inclusion criteria based on Population, Exposure and Outcome, but not Comparator – (i.e., article on marine ecosystem and ES but without evidence of spatial or temporal differences), the article was excluded (Table 2). Because we were targeting primary studies with ES values, we did not consider documents on methods, reviews or on policy analysis without defined ES values in the studies. The full-text screening fit the previous criteria and also considered whether qualitative or quantitative ES values of marine ES and disservices were present. Thus, review papers without ES values or review papers only with ES values from other papers without new analyses were not included.

Articles relating to aquaculture formed a special case in the selection process. The majority of articles related to aquaculture tested technical improvements to enhance the provision of the service of food provision and not the effects of changing environmental conditions. Regarding the eligibility criteria for the full-text screening, most articles on aquaculture were excluded and only articles corresponding to two contrasting situations were selected: (1) when aquaculture was a driver of change of the marine ecosystems and affected the delivery of another marine ES (e.g., impact of pollution generated by fish farming which impact specific ES); (2) when aquaculture was the provisioning service affected by a driver of change of the marine ecosystem (e.g., oyster farming exposed to eutrophication).

##### Study validity assessment

The validity of evidence was not assessed in this systematic map, but information was coded regarding study design elements that may provide some preliminary indication of internal validity. Also, ‘bibliographic content’ was coded with categories of study, review and meta-analysis. Articles producing primary data were coded as such. This information is not intended to provide a comprehensive assessment of study quality, but to highlight details on study type.

##### Data coding strategy

The metadata from all included articles were coded in a standardized data extraction form. The metadata is detailed in a codebook sheet in Additional file 6. For each article, we extracted information on (1) bibliographic information; (2) ecosystem type, specific ecosystem, and biodiversity; (3) ecosystem service; (4) spatial scale of the study, location of the study, temporal scale of the study; (5) driver type, management type; and (6) data type and study design.

The coding was undertaken in three steps.

First, coding was tested on three articles by three reviewers (CSC, LAR, ET) during a face-to-face meeting. This meeting ensured that each reviewer understood the metadata and refined the metadata and its categories when necessary.

Secondly, two reviewers (CSC, LAR), each separately coded a test sample of 30 articles, and compared their extracted data interpretations. Differences were discussed and new adjustments were made when needed. Note that differences only occurred in terms of the way in which to code metadata and how to deal with ambiguous articles.

Finally, CSC and LAR coded all 653 articles, with ET cross-checking specific articles identified as difficult to code. We strove to avoid interpreting information in the article, and concentrated on extracting raw information. To verify consistency throughout the whole coding process, LAR coded a sample of 25 articles twice, at the beginning and at the end of the coding process. Cohen’s kappa coefficient was 0.99, confirming consistency.

##### Data mapping method

The database was managed and analysed in Microsoft Excel software and compiled in one file presented in Additional file 6. The database was analysed quantitatively using tables and graphs like pivot tables and histograms. The identification and prioritisation of key knowledge were done first on the key elements i.e., the ecosystem services, the ecosystem types and the drivers of changes. Bar charts and heat maps were created to provide comprehensible results and show knowledge gaps and clusters on these three elements. We then looked at all others coded information (Table 2) and reported in the present paper information relevant for its novelty or difference with already published information.

While many representations were done in Microsoft Excel, we also used MapChart (https://www.mapchart.net/world.html) for the world map.

Once coding was completed, we checked that our map was a list of publications (i.e., the formats in which authors present their research) all containing only one study unit (i.e., one unique investigation) following James et al*.* [54]. Nevertheless, an article may be classified across several categories of the metadata. For example, an article may involve several ecosystems and/or several ES, but was still one study unit because it was one unique investigation [54]. Consequently, the total number of articles in the different categories of metadata in the results section may be greater than the number of selected articles.

The database contained the mention “unknown” if information was not given by the authors, and “NA” if the coding information was not applicable.

#### Review findings

##### Review descriptive statistics

**Fig. 1:**
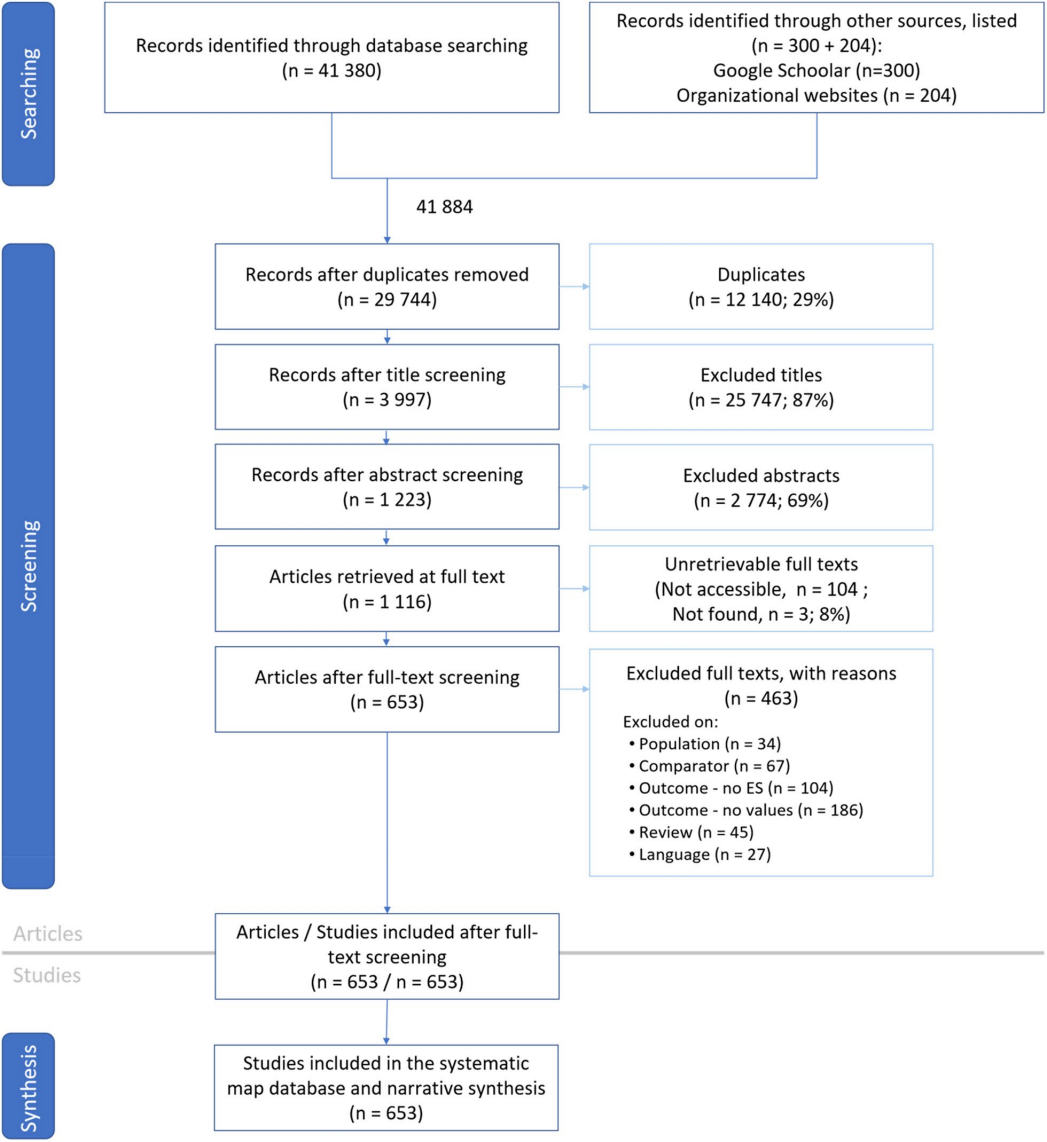
ROSES flow chart for the systematic map showing the number of records included at each stage of the review process

The number of records selected at each stage of the review process is presented in Fig. 1. A total of 41 380 records were identified through database searches, and 504 additional records were identified through Google Scholar and organizational websites. We detected 12 140 duplicate records. The titles and the abstracts were screened separately, resulting in the removal of 25 747 and 2774 records, respectively. The full texts of 1116 records were screened; 107 full texts were unretrievable (listed in Additional file 4).

Full-text screening led to the exclusion of a further 463 articles (listed in Additional file 5). The main reasons were the lack of ES values in the articles or the lack of ES assessment (cf. eligibility criteria). For instance, even if a title or abstract mentioned an ecosystem service, the object of the assessment was often not about an ecosystem service. Similarly observed by Storie et al*.* [38], several articles mentioned the term “ecosystem services”, but did not mention what kind of services were provided/involved. Other reasons for exclusion were, in the order of the number of articles excluded: lack of spatial and/or temporal differences (Comparator); review articles either without ES values altogether, or presenting only existing ES values from other articles without new analyses; missing marine ecosystem (Population) and full text not in English (“Language”) (Fig. 1).

Finally, a total of 653 full texts were selected for coding and are listed in Additional file 6.

##### Descriptive information

1. Bibliographic information

**Fig. 2:**
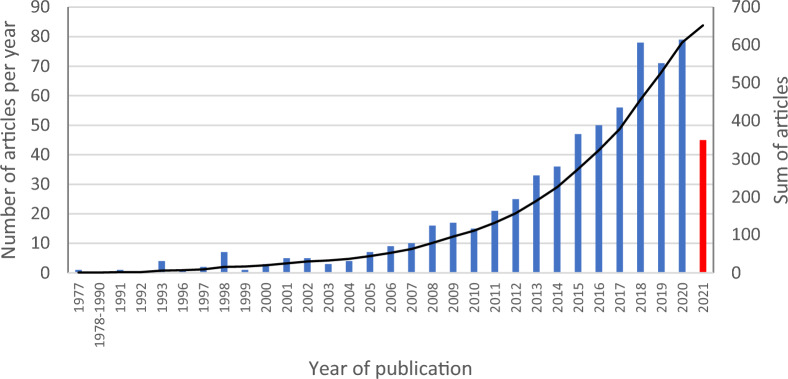
Temporal trend of the number of published articles (no selected article was published between 1978 and 1990), with the number of articles published per year in blue (2021 is shown in red to indicate the year is incomplete: literature search was conducted in July) and the black line shows the increase in number of articles published

The ultimately selected articles covered a period from January 1977 to July 2021 (date of the records searched) with an increase in the number of articles published during the last 20 years (Fig. 2). This trend has been highlighted in many reviews (e.g., [55]), being correlated with the increase of articles published in all fields. A similar pattern was revealed in the temporal evolution of the number of published articles in the 41 380 records identified through database searching (Additional file 7: Fig S1). The increase in studies on ES has already been reported in McDonough et al*.* [56], noting an increase in the number of articles published each year citing the term ‘‘ecosystem services” in the title, keywords or abstracts between 2005 and 2016.

Incidentally, all selected records were journal articles, except one that was a technical report. Although we thoroughly searched the grey literature, only one record met all eligibility criteria. In terms of content, four articles were reviews and one article was a discussion paper. No book chapters or other types of content were included in the final database of documents (e.g., meeting abstracts, news, editorials, commentaries, correspondence, communication, etc.).

The Atlantic Ocean was the most studied ocean (290 articles), followed by the Pacific Ocean (187 articles) and the Indian Ocean (107 articles). The Arctic Ocean was included in only five studies and no study was in the Antarctic Ocean in the selected articles.

**Fig. 3:**
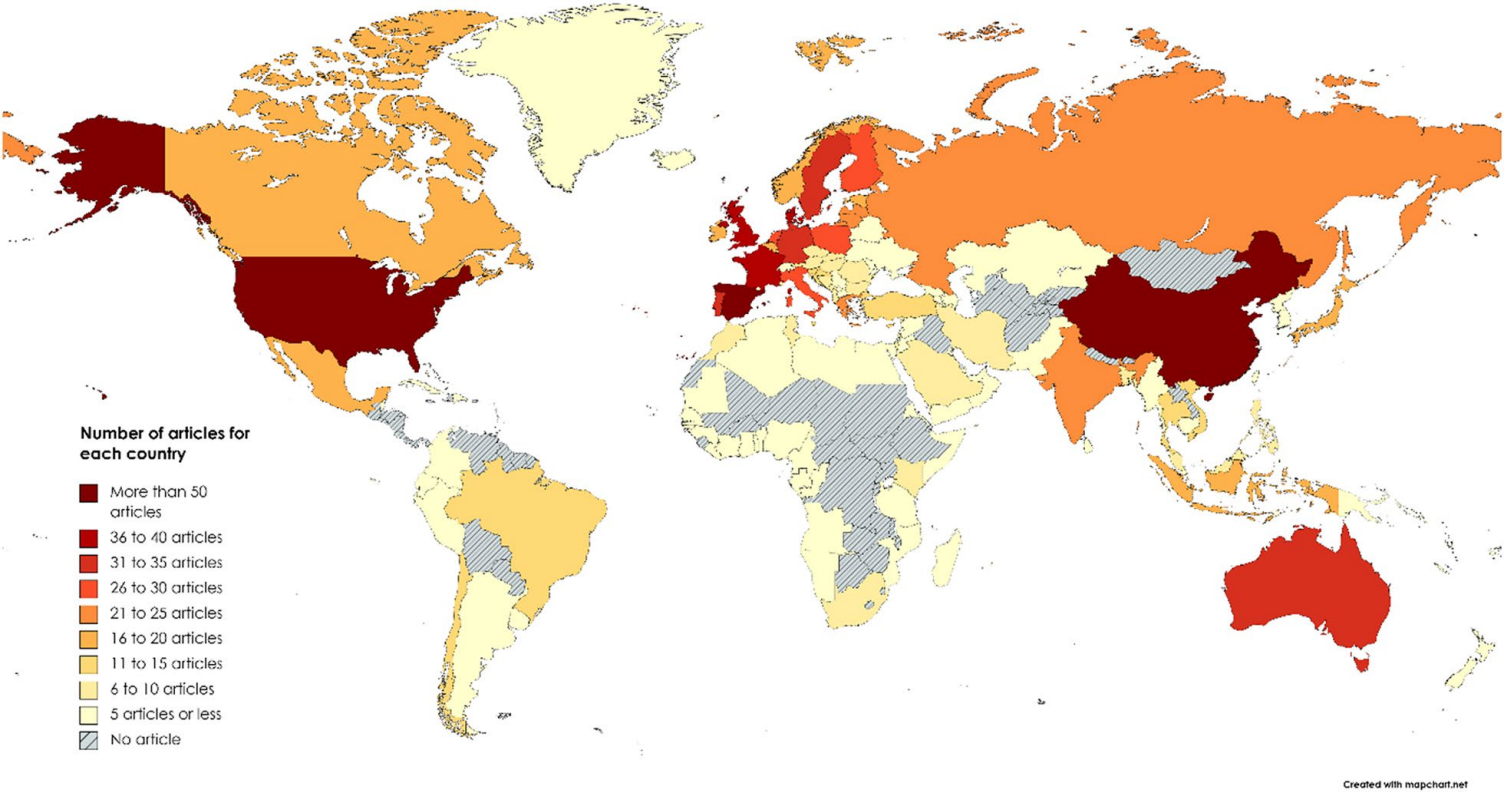
Spatial distribution of the number of articles per country

Study location was coded with the country identified in the articles and related to the study sites presented in the articles. If the article presented a global analysis without a related country, we coded it as “global”. If no study site was mentioned, we coded it as “No case study”. The USA presented the highest number of articles (79 articles), followed by Spain and China (53 and 52 articles, Fig. 3). The USA and China were also in the top three countries along with the United Kingdom (UK) for the highest number of published articles (2005–2016) containing the term “ecosystem services” in the McDonough et al*.* [56] analysis and in a review (1998–2017) on water ES (ref. [55]). While the UK was the fifth country in terms of number of articles in our map, Spain seems to actively publish articles on marine ES, particularly in light of our results and compared with those of McDonough et al*.* [56] and Aznar-Sánchez et al*.* [55].

In this map, we observed a high number of articles involving North America, Europe, Asia and Australia, but few or none in the countries of South America, Africa, the Middle East and Oceania (except Australia). These results follow a trend similar to the global distribution of valuation articles observed in McDonough et al*.* [56] and more recently in the IPBES Values Assessment reports [10], which showed the highest number of articles to be from Europe, North America, and then Asia.

Changes in ES services were analysed mainly at subnational scales, with 61% of the articles (399 articles). Only 16% of the articles (104 articles) involved studies at a national scale, 15% (100 articles) at a supranational scale, 2.5% (16 articles) at a continental scale, and 9% (56 articles) were at the global scale. Again, these proportions, in terms of the spatial scale of the analyses, follow a pattern similar to that highlighted in IPBES [10], which showed 72% of subnational-scale articles, 11% at national scale, 9% at cross-regional/national scales, and 6% at the global scale. Liquete et al*.* [22] also showed a relatively high proportion of local (i.e., subnational) marine and coastal studies.

2) Population: studied ecosystems and biodiversity indicators

**Fig. 4:**
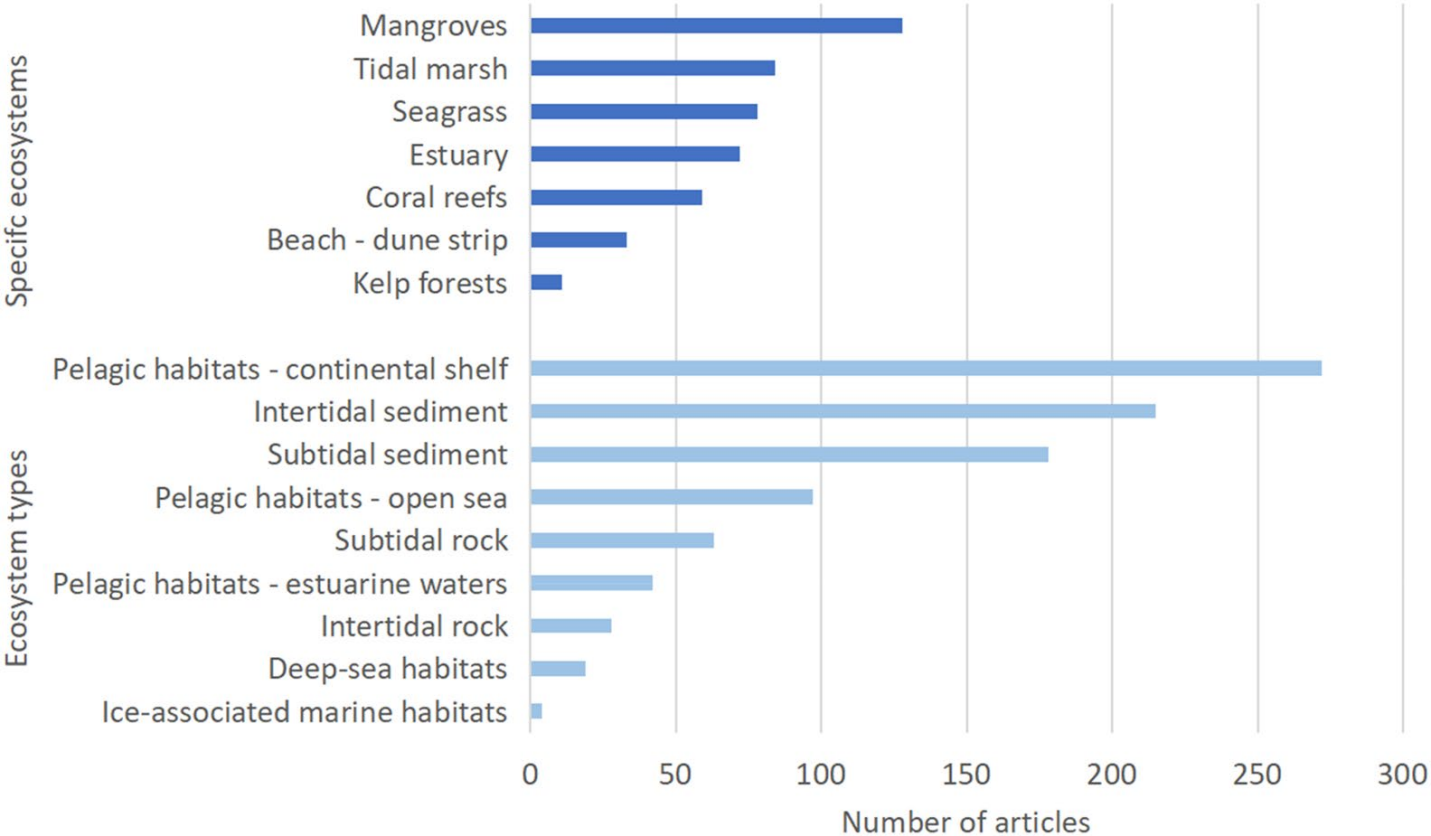
Distribution of articles according to specific marine ecosystems (in dark blue) and ecosystem types (in light blue)

The main ecosystems studied (categories adapted from the “EUNIS level 2 Classification” by the European Commission) were pelagic ecosystems on the continental shelves, and intertidal and subtidal soft-sediment ecosystems (Fig. 4). Few articles dealt with intertidal and subtidal hard substrates and the fewest retrieved articles addressed deep-sea ecosystems and ice-associated ecosystems.

About half of the articles (49%) focused on specific coastal ecosystems (e.g., mangroves, seagrass) (Fig. 4). This focus on specific ecosystems (also called remarkable habitats) has been already highlighted in France [15] and these particular habitats are the subject of disproportionally research studies (e.g., [57]). In these specific ecosystems, mangroves have received the most attention (20%) followed by tidal marshes and seagrass meadows (13% and 12%, respectively). Surprisingly, coral reefs were featured in only 59 articles. Less attention was given to kelp forests, with only 11 articles (2%).

**Fig. 5:**
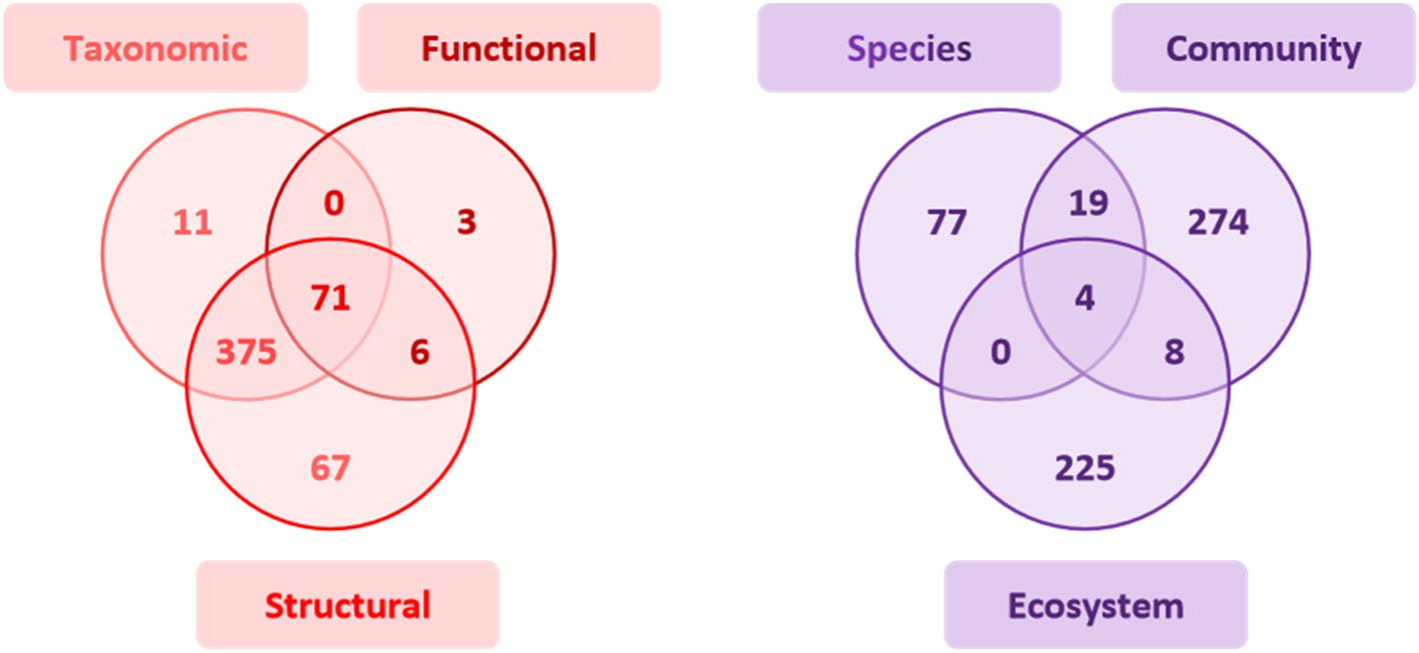
Number of articles per indicator of marine biodiversity monitored per essential biodiversity variable (left panel) and essential characteristic of diversity (right panel)

To describe which facet of marine biodiversity was monitored to depict its changes, we coded essential biodiversity variables (i.e., species, community and ecosystem; cf. Table 2) [58] and the three essential characteristics of diversity (i.e., taxonomic diversity, structural diversity and functional diversity) [44], all detailed in Table 2. Thus, in terms of distribution, community composition [58] was monitored in 302 articles and ecosystem structure in 247 articles, and species’ populations were monitored in 89 of the articles (Fig. 5). The structural diversity (i.e., the distribution of biological entities [44]) and the taxonomic diversity (i.e., the number of different biotic entities like species richness [44]) were the main characteristics of diversity analysed in 375 articles (Fig. 5). Note that not all articles included marine biodiversity elements, so the total in Table 5 is less than the 653 analysed articles.

A qualitative description of the species studied highlights that some charismatic species are often studied, including exploited fish and shellfish species, such as cod *Gadus morhua*, red mullet *Mullus surmuletus* and Norway lobster *Nephros norvegicus*, and foundation species such as mangrove species *Avicennia marina* and *Avicennia germinans,* and the seagrass species *Posidonia oceanica* and *Zostera marina*.

3) Outcomes: ecosystem services

**Fig. 6:**
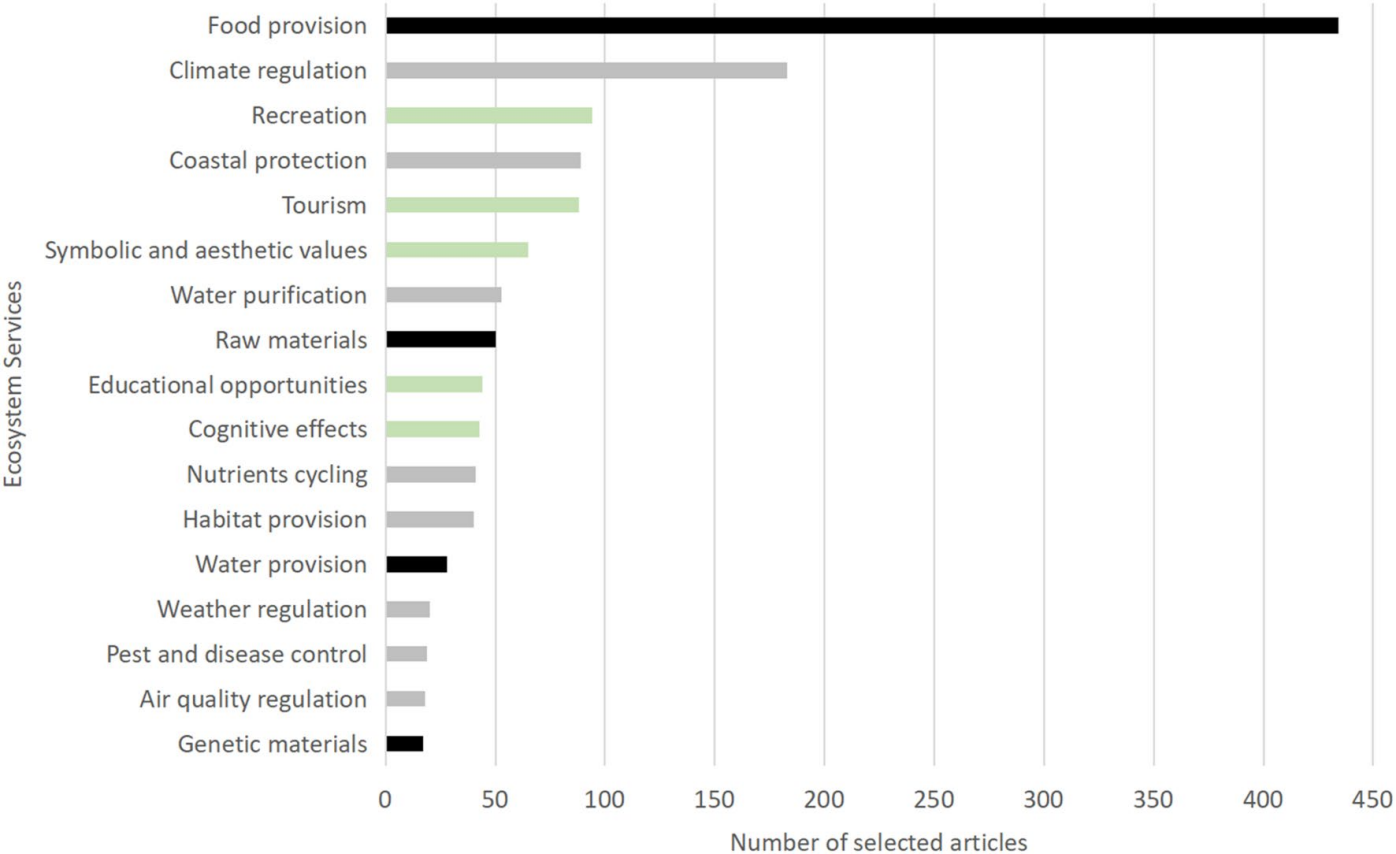
Distribution of the number of articles per ecosystem service. Provisioning services are shown in black, regulating services in grey, and cultural services in green

Provisioning services were assessed in 68% (447 articles), regulation services in 39% (252 articles) and cultural services in 18% (120 articles) of the articles. The main ES studied was food provision (67%; number of articles in Fig. 6) mainly related to fisheries, followed by climate regulation, with 28% of the articles. Recreation and coastal protection were the subject of 14% of the articles, respectively. The least analysed ES were pest and disease control, air quality regulation, and genetic materials with less than 3% of the articles. Only five articles included disservices (i.e., negative impacts on human well-being; for example, related to the proliferation of harmful species like jellyfish [59]). Over time, the literature has focused first mainly on food provision, then progressively covering all the different ES since 2007 (Additional file 7: Figs S3, S4).

The ES are mainly assessed through the potential, capacity or the supply component (89%; number of articles in Table 5), followed by use or flow, which were assessed in 45% of the articles. Preferences, desires, benefits or other forms of demand were assessed in only 8% of the articles. Over time, the proportion of articles considering ES use or flow varied, stabilizing at around 30% during the last decade, during which the number of articles has increased (Additional file 7: Fig S5). While 57% of the articles assessed only one ES component, 42% assessed two components, which were mainly in a “supply/use approach”. Only three articles assessed the three ES components simultaneously.

In the different ES categories, potential, capacity or supply was assessed in between 94 and 100% of the articles, except for the ES food provision, in which they were assessed in only 85% of the articles (Table 5). The ES food provision was assessed through use or flow in 68% of the articles (293 articles), which is different from all the other ES for which use or flow was only assessed in 20% or less of the articles (Table 5). The demand component was also heterogeneously assessed, involving more than 20% of the cultural ES, water purification and air quality regulation, but only 7% and 8% articles on food provision and climate regulation and 10% of articles on weather regulation and nutrient cycling. All ES showed a higher proportion of articles on their benefits than on preferences or desires except cultural services of cognitive effects and educational opportunities.

Following the ES definitions and indicators presented in the articles and their individual definitions, 79% of the articles analysed only one ecosystem service (516 articles). The number of articles decreased with the number of ES identified in the articles, with 7% of the articles (47 articles) analysing two ES and only 7% of the articles (46 articles) analysing more than five ES.

The ES were almost always assessed using biophysical values (91% of the articles, Table 5). Economic values of ES were assessed in 30% of the articles. They were measured using socio-cultural values in only 3% of the articles. Over time, the proportion of articles considering ES economic values varied, stabilizing at between 17 and 31% during the last decade, during which the (absolute) number of articles increased (Additional file 7: Fig S6). The assessment of sociocultural ES values started only in 2006 based on our selection of articles.

Biophysical assessments of ES dominated the assessment of ES in the Baltic Sea (47.5% of articles in [39]). The IPBES report [42] showed that 50% of studies are based on a biophysical assessment, 26% on a monetary assessment and 21% on a socio-cultural approach.

Biophysical and economic ES values were jointly assessed in 21% of the articles. A small number of articles combined sociocultural and biophysical values (5 articles); economic and sociocultural values (4 articles) or combined all three assessment methods (6 articles). We agree with Kuhn et al*.* [46], that “the predominant focus on biophysical research is emphasized by the fact that the vast majority of publications is focused on ES supply, neglecting the demand side and leaving out the societal request for ES”.

Although biophysical value was assessed for all types of ES, the economic and socio-cultural values were more common for some specific ES (Table 5). For instance, economic values were frequently assessed (more than 70%) in articles on raw materials, genetic materials, and air quality regulation. Sociocultural values were considered for 26% and 24% of the ES related to pest and disease control and genetic materials. The economic and socio-cultural values were the least frequently assessed values for the ES climate regulation, food provision, and nutrient cycling.

4) Comparator: spatial and temporal scale

In our map, the spatial scale of the analysis of ES changes was measured using the number of case study sites. For instance, 247 articles involved one site (37% of the articles), 275 articles analysed more than one site (41%) and 187 articles (28%) more than three sites, with a maximum number of sites (536 sites) in a study on coastal tourism under climate change on beaches all over Japan [60].

Temporal dynamics were coded with the interval and the time covered by the raw and the results data. While we did not consider articles with data covering periods ending before 1900 (cf. eligibility criteria), data acquisition varied from 1 to 2500 years, e.g., from 500 BC to 2000 in Finney et al. [61]. A large majority of articles (83%) covered a period of more than 1 year (Additional file 7: Fig S2). The duration of the period studied was longer in the results data, because the raw data were used in simulation models, i.e., for prediction. A total of 170 articles (26%) studied more than one site with data covering more than a year.

In terms of the study period, 490 articles (76%) analysed data from the past (i.e., prior to 3 years before the date of publication), and 446 articles (69%) reported the situation in the last 3 years before publication and 146 articles (22%) analysed services in the future (i.e., after the year of publication).

5) Drivers of change

**Fig. 7:**
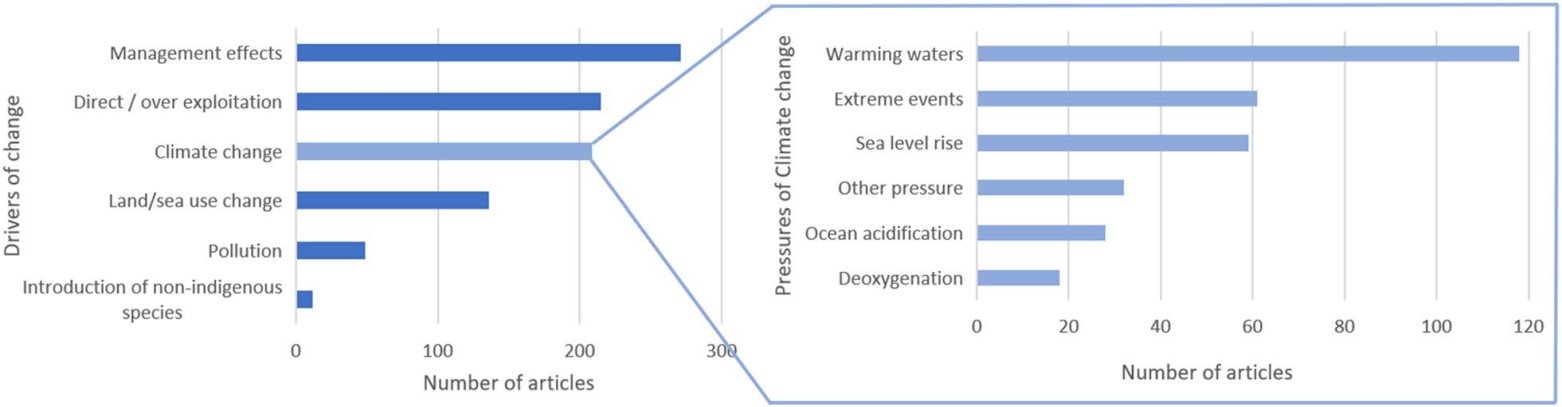
Distribution of the number of articles for the types of drivers of change (on the left) with distribution for the pressures related to climate change (on the right). Article can concern several drivers of change or pressures of climate change

Coastal and marine ecosystems are affected by several drivers of change, which in turn affect the delivery of marine ES [26]. About 60% of global marine ecosystems have been degraded or unsustainably used [49], and the percentage of stocks fished at biologically unsustainable levels has increased from 10% in 1974 to 34.2% in 2017 [63]. Within the six coded classes of driver types (Fig. 7), 48% of the articles (315 articles) identified only one driver and 38% (247) identified more than one driver, 14% did not identify or mention a driver of change at all. Finally, 58% (376 articles) integrated data regarding drivers of change into their analyses and 29% (187) integrated data from the ecosystem condition or processes into their analyses.

Within the different coded types of drivers of change, the management effect was the most analysed driver (41%; Fig. 7), followed by direct/overexploitation, analysed in 32% of the articles. Climate change was analysed in 31% of the articles and land/sea use and change in 21% of the articles. In terms of climate change pressures, warming waters was the most analysed driver. The introduction of non-indigenous species and deoxygenation (related to climate change) were the least frequently analysed pressures.

Regarding management, fishery management concerned 33% of the articles, water quality management 6% and finally marine protected areas, 9%.

6) Data and study types

Almost all articles were based on quantitative data (99%; 645 articles); qualitative data were exploited in 4% of the articles. The dominance of quantitative data is also highlighted in Liquete et al*.* [22], reporting 56% of quantitative assessments, 10% of qualitative assessments and 16% of mixed analyses.

Within the different ES, qualitative data primarily addressed cultural services, accounting for 13 to 19% of the articles (Additional file 7: Table S1). Overall, 57% of all articles presented primary data with the fewest primary data articles for the food provision and genetic materials ES (respectively 49% and 53%). With a view to carry out a meta-analysis after the systematic map, the presence of measures of variability, such as standard errors or standard deviations of ES values, was coded: information on variability was provided in 58% of the articles.

**Fig. 8:**
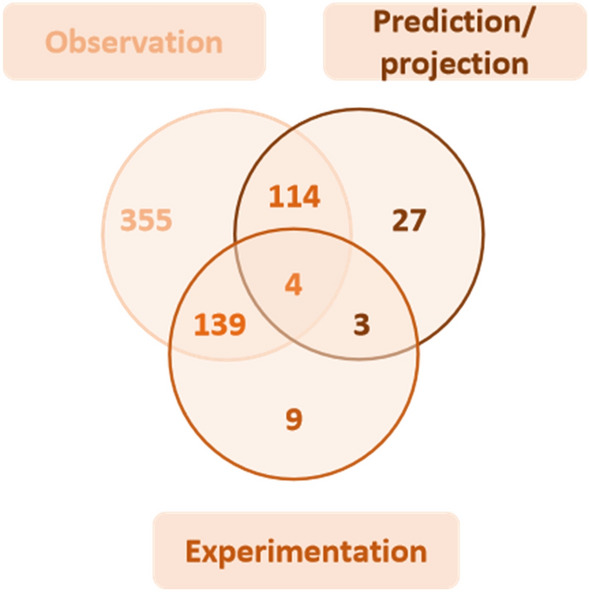
Number of articles per type of data

The data were mostly based on observation and descriptive approaches with measurement of a specific parameter (92%, 598 articles), representing 100% of the articles on genetic materials, water provision, air quality regulation, weather regulation, and pest and disease control (Additional file 7: Table S1). Projection or prediction approaches (definition of potential values in the future based on models; projection is future when a change/pressure happens; prediction is future when nothing influences the evolution) were used in 22% of the articles (146 articles) and experimentation (experiments showing causality effects between factors) was used in 23% (153 articles). A mixture of observation, prediction or projection, and experiment data was reported in four articles. Experimentation alone was present in 9 articles (Fig. 8).

**Fig. 9:**
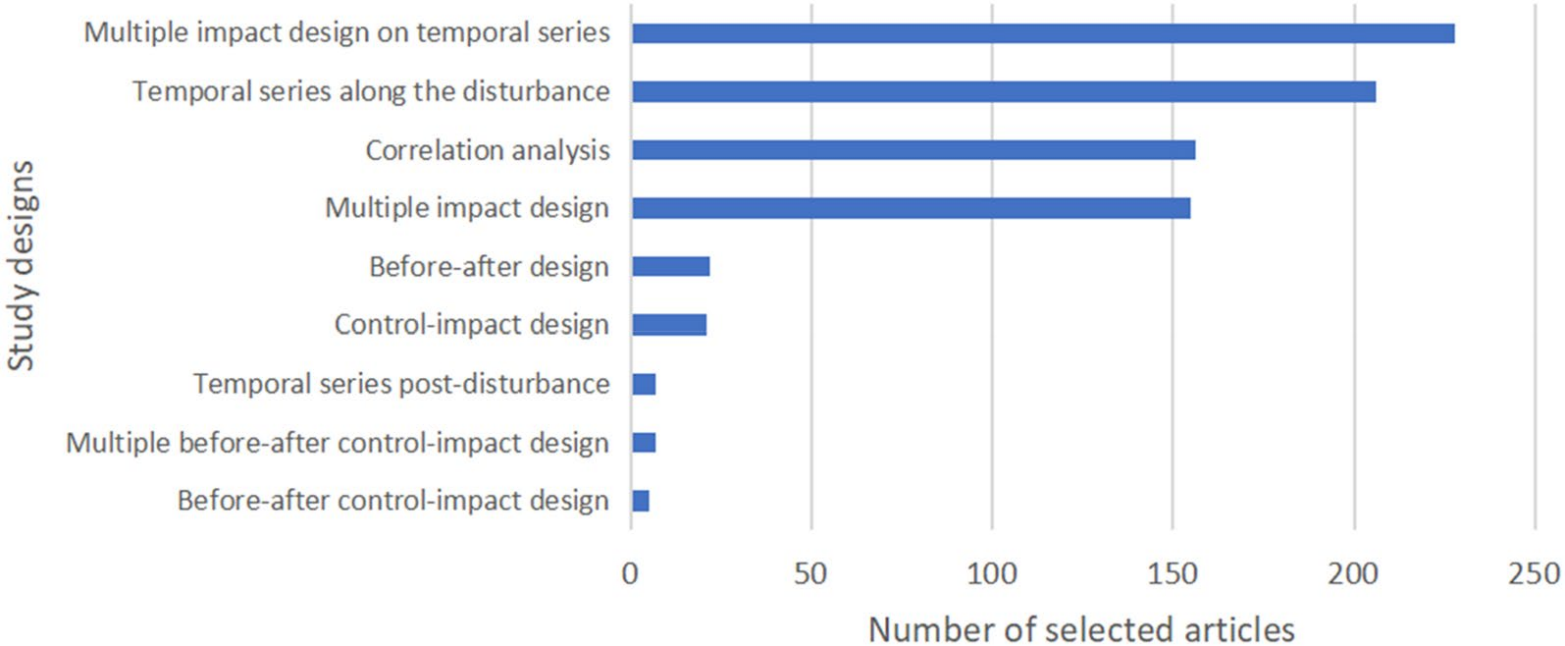
Distribution of the number of articles for the types of study design across case studies

The main types of study design were multiple impact design on temporal series, which refers to two or more ecosystems/areas/species with different characteristics compared over time (35% of the articles, Fig. 9). Temporal series during a disturbance (i.e., one ecosystem/area/species or several studied over time during a disturbance) followed, with 32% of the articles. A multiple impact design (i.e., two or more ecosystems/areas/species with different characteristics to compare at one time point) was used in 25% of the articles. Correlation analysis between drivers and one or several ecosystems/areas/species was provided in 24% of the articles. The study design with analyses before and after an event or sudden driver of change were the least studied.

Within the different ES, the proportion of the different study types was homogenous with the mean of all ES (Additional file 7: Table S1), except for climate regulation, nutrients cycling, habitat provision, pest and disease control, symbolic and aesthetic values, recreation and tourism which were assessed more frequently in studies with a multiple impact design (two or several ecosystems/areas/species with different characteristics to compare at one time).

7) Cross-category analyses

The number and proportion of articles on the different ES showed a similar pattern for the different marine ecosystems, with the intertidal sediment and subtidal sediment ecosystems being the focus of most articles (Table 6). An exception was articles on food provision, which especially involved pelagic habitats on continental shelves. For the specific marine ecosystems, mangroves attracted the most articles examining the various ES. However, estuaries and tidal marshes had proportionally more articles on air quality regulation. Articles on cultural services account for around 20% of the articles on beach—dune strip, mangroves and coral reef.

The coded biodiversity indicators showed similar patterns within the ES (Table 6). Ecosystem structure was the most monitored biodiversity indicator across all articles on different ES, except for food provision which was particularly studied in terms of community composition. Structural diversity and taxonomic diversity [44] showed similar patterns within the different ES. Functional diversity, which is the diversity of functions or functional traits, was generally the least studied across all ES.

The heat map on Table 7 demonstrates that 48% of the articles on food provision studied the impacts of management effects and/or direct/overexploitation. For the other ecosystems, the impacts of land/sea use change were most studied, involving 29–64% of studies depending of the ES. Nutrient cycling and coastal protection were relatively more frequently studied in relation to climate change impacts (39 and 48%). Regarding the specific climate change-related pressures, warming waters and sea level rise were the focus of most articles, with extreme events supplanting either of these top two pressures or coming in at a close third place for the articles on raw materials, water provision, coastal protection, weather regulation, habitat provision and pest and disease control (Table 7).

**Table 5:**
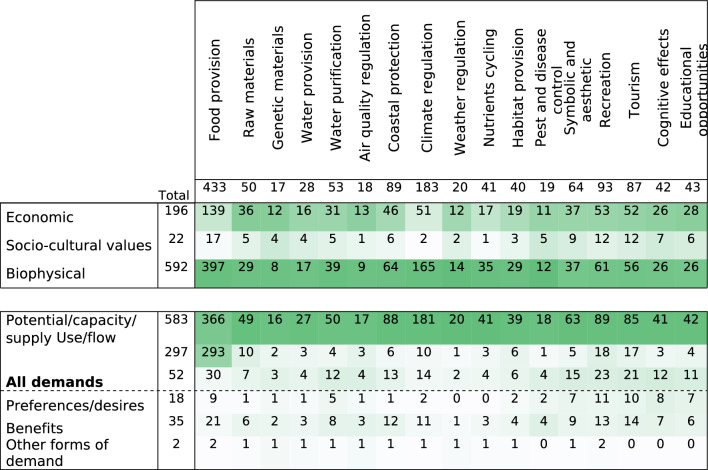
Distribution of the number of articles per ecosystem service values and components (cells are shaded according to the high (dark) and low (light) values for each column separately)

**Table 6:**
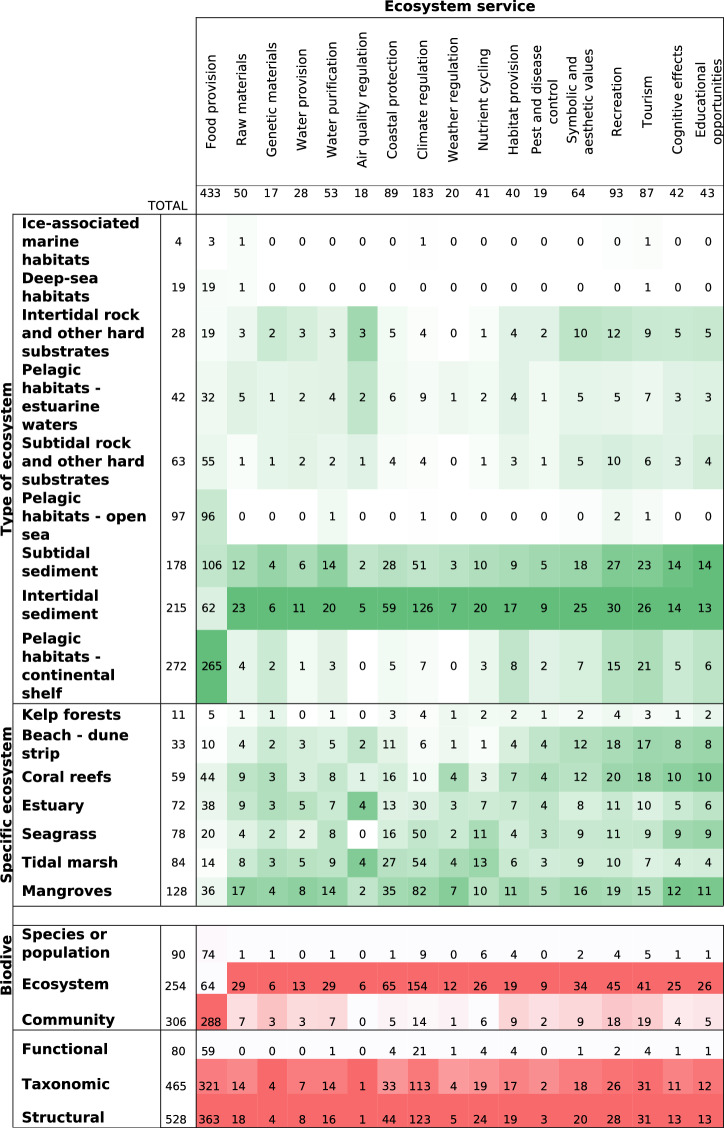
Distribution of the number of articles per ecosystem service, ecosystem type, and biodiversity component (cells are shaded according to the high (dark) and low (light) values for each column separately)

**Table 7:**
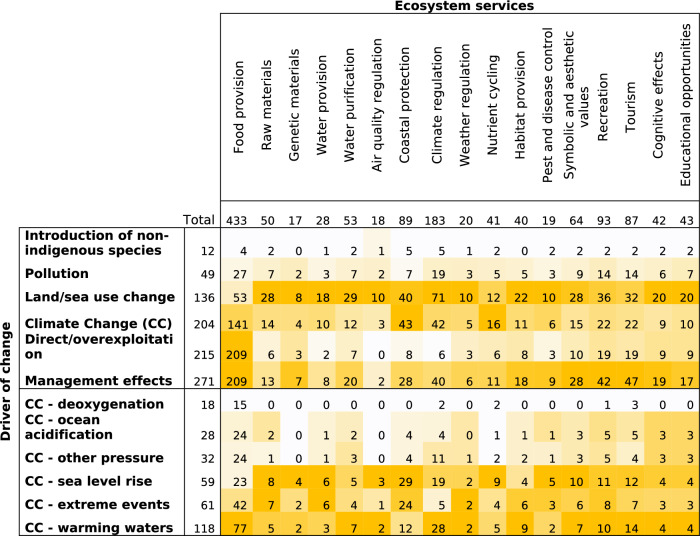
Distribution of the number of articles per ecosystem service and type of driver of change (cells are shaded according to the high (dark) and low (light) values for each column separately)

##### Comparison with other evidence syntheses

To our knowledge, no other systematic map has been published on the evidence of how ecosystem service delivery is affected by changes in marine ecosystem structure and functioning. Nevertheless, evidence syntheses published on related subjects were used to compare our map results. The final number of analysed articles (653) is close to that reported for maps on the impact of agroforestry on ES and human well-being in high-income countries [64] and on the analysis of publication trends on water ES [55], but higher than other evidence syntheses on related subjects (Table 8). Our number of articles is low compared with the review of the overall ES literature [65], i.e., not restricted to marine ecosystems and their dynamics.

**Table 8:** Comparing other evidence syntheses to our current map

Citation	Scope of review	Nature of synthesis	Search databases	No. of other literature sources	Publication date range of included articles	No. of included articles
Our systematic map	Impact of changes in marine ecosystem structure and functioning on ecosystem service delivery	Systematic map	3 (WOS, Scopus, Google Scholar)	6	1977 to 2021 (July)	653
Castle et al. [64]	Impacts of agroforestry on ecosystem services and human well-being in high-income countries	Systematic map	5 (WOS, Scopus, EBSCO: Agricola, Econlit, CAB Abstracts and Global Health, AGRIS)	24	1990 to 2020 (June)	632
Inácio et al. [65]	Mapping lake ecosystem services	Systematic review	3 (WOS, Scopus, Google Scholar)	0	2000 to 2021	30
Storie et al. [38]	Impact of Baltic Sea ecosystems on human health and well-being	Systematic map	17	7	1975 to 2020	67
Aznar-Sánchez et al. [55]	The worldwide research trends on water ecosystem services	Bibliometric analysis	2 (WOS and Scopus)	0	1998 to 2017	782
McDonough et al. [56]	Analysis of publication trends in ecosystem services research	Bibliometric analysis	4 (Scopus, WOS; CABI: CAB Abstracts, and Environmental Sciences and Pollution Management)	0	2005 to 2016	Approximately 3000
Liquete et al. [22]	Current status and future prospects for the assessment of marine and coastal ecosystem services	Systematic review	1 (SciVerse Scopus)	0	1823 to 2012	145

*WOS* Web of Science

We analysed more articles than Liquete et al*.* [22], likely due to the publication date range: we considered all articles up to July 2021 and Liquete et al*.* considered articles only up to 2012. Our selection of articles from this 8 year interval contains 496 articles. Thus, our database up to 2012 contains 157 articles, a figure close to the 145 articles considered in Liquete et al. [22].

#### Limitations of the map

##### Limitations in searching

The search string and the articles accepted were only in English. Like for most of the maps or reviews, this restriction biased the distribution of the articles, with around 30% of the articles coming from English-speaking countries, as reported in Collins et al*.* [66]. Integrating an additional language (e.g., French or Spanish) would have increased the range of the map, but also introduced other potential biases by focusing on some countries at the expense of others; an exhaustive search should ideally include all or the mainly used languages around the world but we did not have the resources or the time to integrate additional searches in other languages.

While the searches obviously depend on the search terms and the databases used, we adopted a comprehensive approach to limit this dependency.

##### Limitations in screening

The kappa coefficient at the title screening step was calculated on only 2.38% instead of 10% of titles given the high number of records (29 744 records) screened at the title step. Due to resource and time limitations, we chose to screen 1001 records by two screeners. The CEE recommends pilot testing on 10%, which is considered as the necessary proportion to thoroughly test and ensure that criteria are correctly defined so that no relevant evidence is missed during screening. Although we were not able to abide by this guideline, we carried out a thorough training phase and applied a conservative approach during all screening steps. In addition, we chose to apply relatively strict criteria at the abstract screening stage, based on the absence of the Comparator items. This pragmatic decision was taken in light of the very large volume of literature and limited human resources. We conducted a posteriori crosscheck checking if abstracts have information about the Comparator (e.g., information of ES change), which confirmed in principle that abstracts provided the required information.

##### Limitations in coding

The test of the coding procedure highlighted some limits of the coding categories of the protocol [41], such as the difficulty of differentiating a “local” scale of analysis from a “subnational” scale, depending on the size of the study and the country involved. To overcome this limit, we grouped these two levels into a single level (“subnational”) in our analysis. All improvements on the categories coded are detailed in Table 4.

Coding was generally strictly based on the data in the article, but the EUNIS ecosystem classification and the ES classification were coded based on interpretation of the information in the articles. When difficulties were encountered, the reviewers held discussions and reached decisions together. If the same hesitations or difficulty in coding came up more than once, we strove to find overall solutions to apply across the board and maintain coding consistency throughout the analysis.

## Conclusions

This map highlights knowledge clusters and gaps on the impacts of the spatio-temporal dynamics of marine ecosystems and biodiversity on the ecosystem services they provide. A high number of records was identified in our search (29 744 records without the duplicates) with 2.2% (653) selected for the systematic map. This low number of mapped articles can be linked to the frequent use of keywords relating to ES for articles covering very different subjects, a point also highlighted in [38, 67].

We focused on the ES affected by marine ecosystem dynamics, but our map’s results show that 9 years after the well-cited Liquete et al*.* [22] article, similar knowledge clusters and gaps in the marine and coastal ecosystems remain. Nevertheless, some efforts can be highlighted, such as the recent increase in the number of articles on the different values of ES, e.g., ES benefits and preferences.

Our systematic map combines a large amount of information on ecosystems, ES with their values and components, types of temporal and spatial dynamics, drivers of change, study type and data type. Compared with other reviews on marine ES, we introduced new information on marine ES literature, such as the type of study design and the type of temporal and spatial dynamics.

### Implications for future research

Marine ecosystems receive much less attention than terrestrial ecosystems in ES research [37, 68]. In our review of the literature on ES affected by marine ecosystem dynamics, we highlighted differences among articles within the marine ecosystems and the marine ES, revealing different levels of interest and knowledge.

The proportion of articles within the different ES categories in this systematic map with 68% of provisioning services, 39% of articles on regulation services and 18% on cultural services differ from other studies. Systematic maps on marine and coastal ES in the Baltic Sea showed different patterns, with cultural services as the most assessed ES categories [38, 39]. Studies on ES provided by lake ecosystems [65] and on terrestrial ecosystems [69–72] reported that regulation services were the most assessed. Nevertheless, the knowledge gap on marine cultural services has already been highlighted [73] as well as the focus of cultural ES research on land-based assessments [74] which can generally be related to the difficulties identifying and appraising intangible attributes [73], such as aesthetic, symbolic, and bequest values [73]. Also, methods to quantify indicators of cultural services generally only capture a discrete, snapshot value, for lack of measures of changes over time [73], and therefore do not include the dynamics of the marine ecosystems. Recreation and tourism are the most studied cultural services in our map, likely due to their socio-economic importance and the fact they are easier to assess and quantify [73, 75]. Even though the importance of recreation and tourism is unquestionable, other cultural services need to be considered more extensively and assessed [73, 75]. In the different ES components, the dominance of potential/capacity or the supply component (90%, 599 articles) was also observed in Kuhn et al. [39], Inácio et al. [65] and IPBES [10].

Food provision was the most studied marine ES, particularly for fisheries. Our results were influenced by the high proportion of articles on food provision (i.e., fisheries), which is an important ecosystem service that marine ecosystems provide, having high economic importance for humans. Some marine species groups are more frequently assessed and studied such as commercial species and top predator fish stocks [30]. Regarding tourism or recreation, our screening process retrieved literature on the impact of tourism and/or recreation activities on the ecosystems, which we excluded as out of scope. Furthermore, the existing ES analyses have not integrated how the impact of tourism and/or recreation activities on the ecosystems also affects all ES as well as the tourism and/or recreation activities themselves, thus shaping the sustainability of these activities. For example, Apps et al*.* [76] studied how scuba diving can impact the behaviour of the grey nurse shark and Harriott et al*.* [77] studied recreational diving and its impact in marine protected areas in Eastern Australia. However, neither of these studies explored how these impacts affected the sustainability of the recreational activities as a feedback loop.

Knowledge on marine ecosystems decreases with distance from the coastline, as previously shown in [78]. Knowledge clusters are concentrated in the pelagic ecosystems on continental shelves and intertidal and subtidal soft-sediment ecosystems, and less attention has been given to deep-sea ecosystems [37, 79] and ice-associated marine ecosystems [80]. The relatively low volume of ES literature for these latter two ecosystems can be explained by their relatively less accessible habitats. They may also be ecosystems that—by nature—provide fewer ES in terms of diversity and in quantity compared with other marine or terrestrial ecosystems. Deep-sea research incurs high costs, difficulties and risks associated with the ecosystem characteristics [81]. However, deep-sea ecosystems are growing centres of interest for extracting mineral resources [82] and, although some studies have analysed the potential impact of mining on deep-sea biodiversity, research efforts also need to be directed at estimating the potential impact of human activities on their ecological conditions and ecosystem service provisions. Recent publications have addressed the impacts of deep-sea mining on microbial ES [82] and how to incorporate ES into the environmental management of deep-seabed mining [83]. Articles on deep-sea ES highlight many ecosystem “functions” and “support services” such as habitat provision and nutrient cycling [81]. Mangroves are the most studied specific ecosystem, followed by tidal marshes and seagrass meadows, also highlighted by [15], and kelp forests are the least studied. As shown in Jacquemont et al*.* [84], the capacity to provide ES and the volume of papers are not related to the global surface area of the habitat. For instance, in contrast to soft-sediment habitats, mangrove ecosystems provide a high quantity of ES per unit area and have been intensely studied, even though they represent a small surface area on the globe [84]. Among specific ecosystems, macroalgae have received little attention, but current focus is turning to kelp forests in light of the growing interest in blue carbon [85].

Most drivers of change directly affect the ecosystem status and functioning and therefore its ability to provide ES, but management effects may either consist in reducing the pressures or even the very provision for some ES. When effective, management is expected to lead to positive results regarding ecosystem preservation and sustainable ES consumption. Across the different types of drivers of change, management effects, followed by direct/overexploitation and climate change, are the most studied. IPBES [86] has shown that the highest relative impact of direct drivers on the marine realm based in terms of essential biodiversity variables is direct exploitation (management effects are not a category of direct anthropogenic drivers in IPBES), followed by land/sea use change and then climate change. Therefore, the pattern of knowledge clusters closely reflects the relative impacts of the drivers of change. The introduction of non-indigenous species and pollution have the lowest relative impact on the marine realm [86], but it is nevertheless important to grow knowledge on their impact on marine ES given their increasing frequency [68]. The need to develop the knowledge base on the efficiency of management actions in marine ecosystems has been highlighted [15]. Management effects have the highest number of articles within the types of drivers of change so that the database of our systematic map could be used to analyse management efficiency.

Time-series study designs are common, but control-impact and/or before-after designs are the least implemented study designs. This discrepancy can be attributed to the spatial scales at which ES are provided and affected by the drivers of change on marine ecosystems. With regard to the questions raised in our study, the establishment of long-term time series is better suited to the study of ES than the development of experimental approaches or control-impact and before-after study designs. For example, it is difficult to design experiments to follow the responses of fisheries to climate change or overexploitation; in contrast, time-series analyses and prediction or projection are more suitable and more frequently implemented. One interesting perspective is to extend the scope of the systematic map to the feedback loop of ES variation on other ES and on human demand. For example, drivers of change impact marine ES, which affect ES uses, which in turn also affect their sustainability. In addition, the multifunctionality and the bundles of services are not sufficiently studied [15] and have only been rarely studied in marine realm.

This systematic map confirms hypotheses and results on marine ES knowledge presented throughout this paper, although our systematic map focuses on marine ES affected by marine ecosystem dynamics. The database presents detailed information on the knowledge within the ES and ecosystems categories, thereby identifying very specific knowledge gaps for future research. The database can thus be used as a source of articles for a meta-analysis on related topics. As for future prospects for the systematic map defined here, we agree with Collins et al*.* [66] on the interest to explore the use of computer algorithms to construct and update the maps, particularly in light of the high and increasing number of articles to search, screen and code in the systematic map process.

### Implications for policy/management

The ES concept is increasingly used and implemented in policy and management tools, because it is known to increase the consideration of nature and its contributions to people into land or marine planning [68]. This concept is increasingly cited in international and national regulations and policies, but its implementation is challenging, requiring further solid scientific knowledge [68]. Indeed, “*future efforts should be aimed at developing solid evidence linking decisions to the anthropogenic impacts on ecosystems and generated services and, as a consequence, to human well-being; working with leaders in governments, businesses, and civil society to develop and provide knowledge and tools to effectively integrate ecosystem services into decision-making processes; and reforming policies and institutions, and building capacities to better align with private, short-term goals and with societal, long-term goals”* [68].

The lack of knowledge is a danger for the sustainability of human actions and knowledge-based nature conservation. The knowledge gaps and clusters highlighted here have an impact on the beneficial development of policy and management practices. For example, limited evidence on the efficiency of management actions in marine ecosystems has been highlighted [15, 73]. Given that management effects have the highest number of articles among the types of drivers of change coded, the database of this systematic map could be used to analyse management efficiency further. While management actions concerned many fisheries regulations, more regulation needs to be applied. Marine protected areas (MPAs) are a key tool increasingly used for marine protection and conservation [67, 87]. Nevertheless, the number of articles on MPAs in the map is low, despite the growing number of articles over the last 10 years. Studies on MPAs primarily assess the biological responses of their implementation, with less emphasis on the impact of ES delivery (but see the recent review of the ES, societal goods, and benefits of MPAs [67]). There is a need to grow knowledge on the efficiency of MPAs and other conservation actions to better guide their implementation depending on the context, desired level of protection, and conservation targets [68].

The consideration of the plurality of nature’s value is absolutely essential to cultivate a sustainable and equitable future, as recommended by the latest IPBES report [10]. Nevertheless, the economic and socio-cultural values of marine ES are still poorly known and have generated less interest. As funders and/or government authorities, decision-makers can push for more transdisciplinary science and research at the science-policy interface as well as for the participation of different types of decision-makers in research. They can also advocate more studies on the desired and preferred ES that are poorly studied. For example, beach—dune strips present one of the lowest numbers of articles even though they are ecosystems of high importance for local economies through the many recreational and tourist activities they afford and for mitigating numerous anthropic pressures. These conflicts of use have wide political implications and are largely exposed to climate change.

## Supplementary Information

**Additional file 1: **ROSES for systematic map reports

**Additional file 2: **Search strings and comprehensiveness

**Additional file 3: **Test list

**Additional file 4: **List of unobtained full texts

**Additional file 5: **Articles rejected during the full-text screening process

**Additional file 6: **Systematic map database—List of all selected articles with their codes

**Additional file 7: Figure S1** Temporal evolution of all articles before the screening process with the number of articles published per year in blue and the increase number every year in the black line. **Figure S2** Temporal cover by the raw and results data. **Figure S3** Temporal evolution of the number of published articles within the different marine ecosystem services. **Figure S4** Proportion over time of published articles within the different marine ecosystem services. **Figure S5** Proportion over time of published articles on ES components. **Figure S6** Proportion over time of published articles on ES values. **Table S1** Distribution of the number of articles per Ecosystem Service and type of data, study design, time frame and time data (the colour of the cells is set according to the high and low values of each column separately)

## Data Availability

Datasets produced by the systematic map are available as supplementary material.
