# Supplementary material for: Existing evidence on the impact of changes in marine ecosystem structure and functioning on ecosystem service delivery: a systematic map
Source: Environ Evid. 2023 Jul 20;12:13. doi: 10.1186/s13750-023-00306-1 (PMC11378828; doi:10.1186/s13750-023-00306-1)
Supplement: Supplementary file 7 — Additional file 7: Figure S1 Temporal evolution of all articles before the screening process with the number of articles published per year in blue and the increase number every year in the black line. Figure S2 Temporal cover by the raw and results data. Figure S3 Temporal evolution of the number of published articles within the different marine ecosystem services. Figure S4 Proportion over time of published articles within the different marine ecosystem services. Figure S5 Proportion over time of published articles on ES components. Figure S6 Proportion over time of published articles on ES values. Table S1 Distribution of the number of articles per Ecosystem Service and type of data, study design, time frame and time data (the colour of the cells is set according to the high and low values of each column separately) [file 13750_2023_306_MOESM7_ESM.docx]

Figure 1 of AF7: Temporal evolution of all articles before the screening process with the number of articles published per year in blue and the increase number every year in the black line


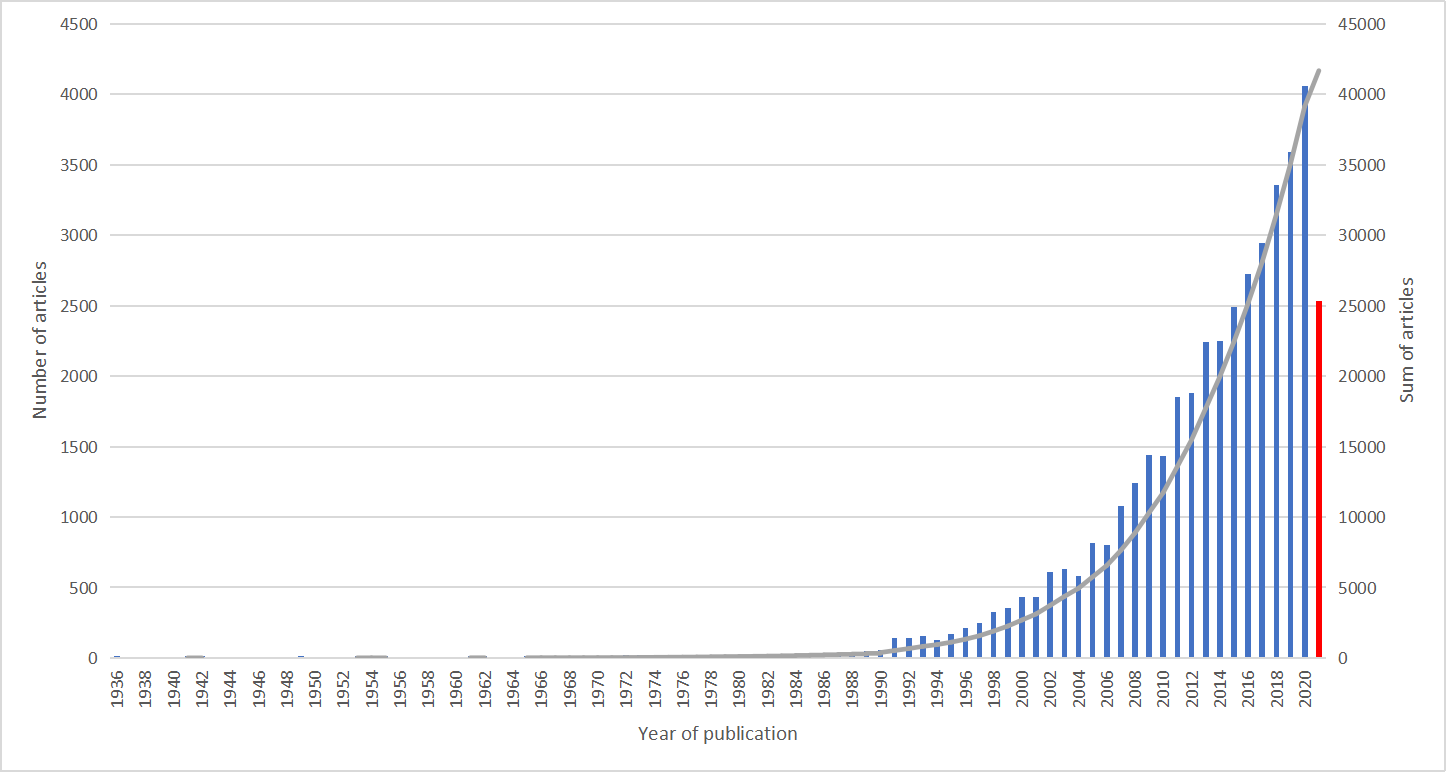


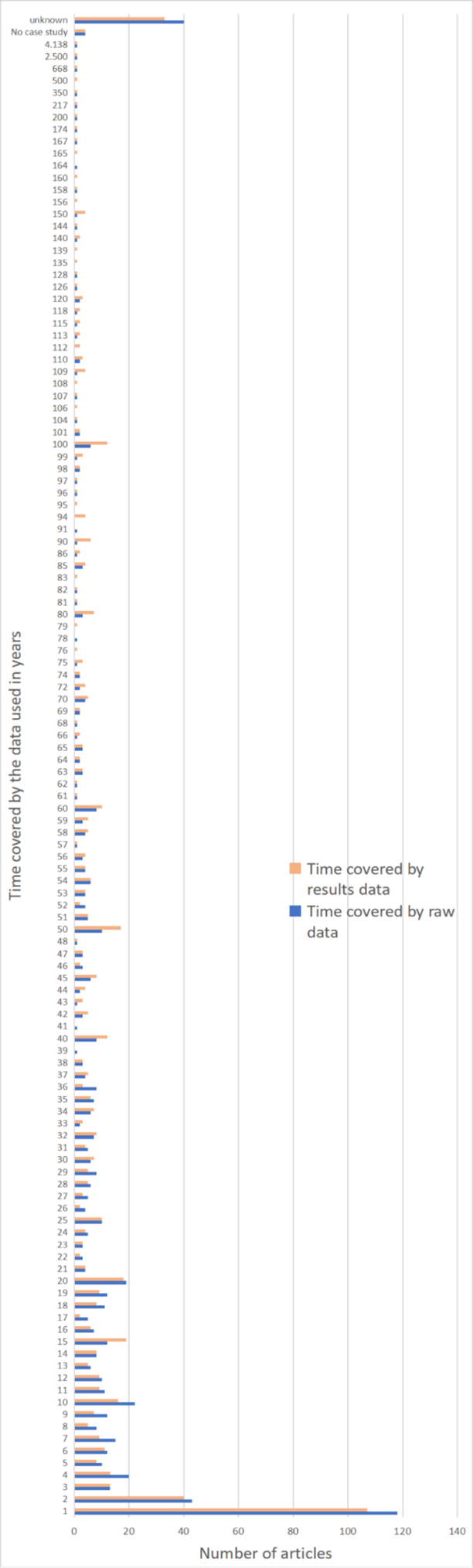
Figure 2 of AF7: Temporal cover by the

raw and results data

Figure 3 of AF7: Temporal evolution of the number of published articles within the different marine ecosystem services


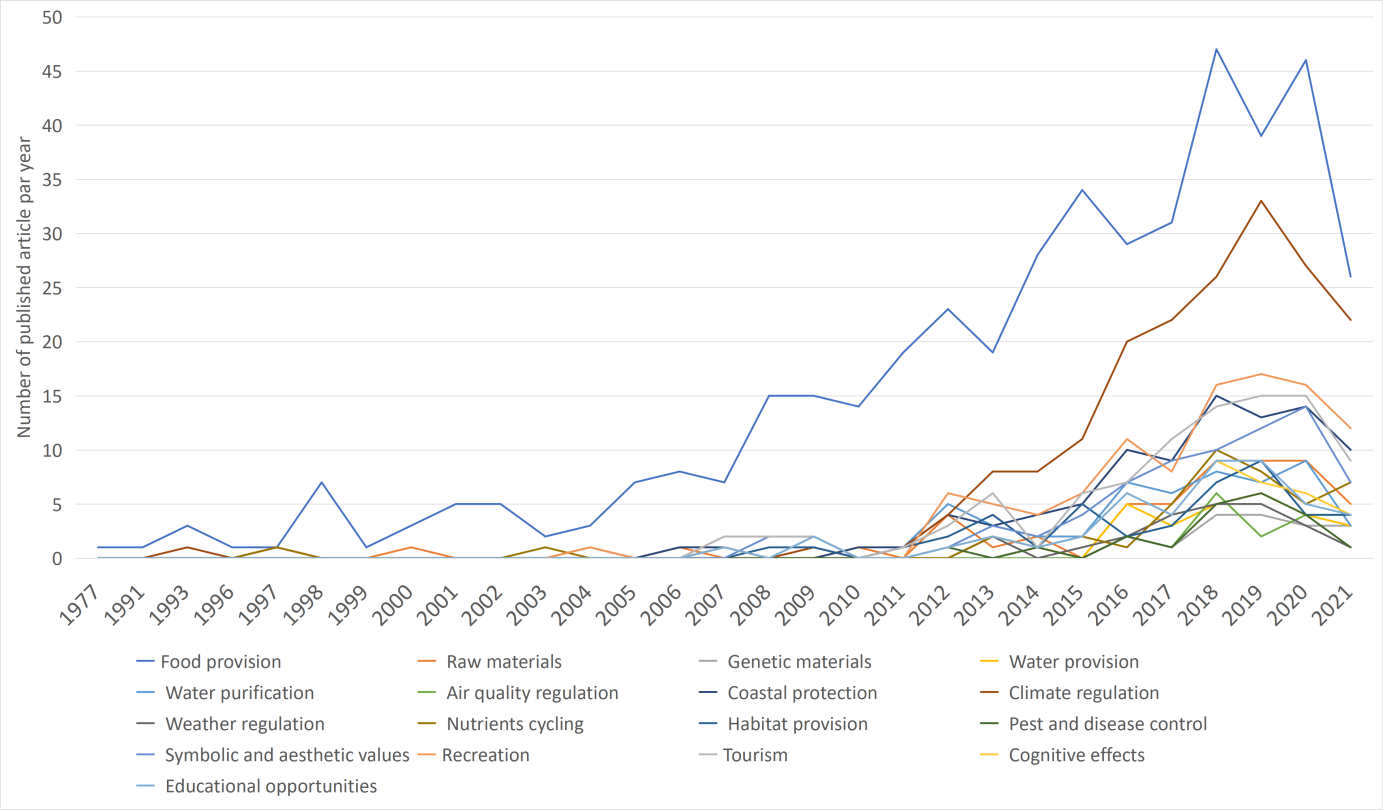


Figure 4 of AF7: Proportion over time of published articles within the different marine ecosystem services


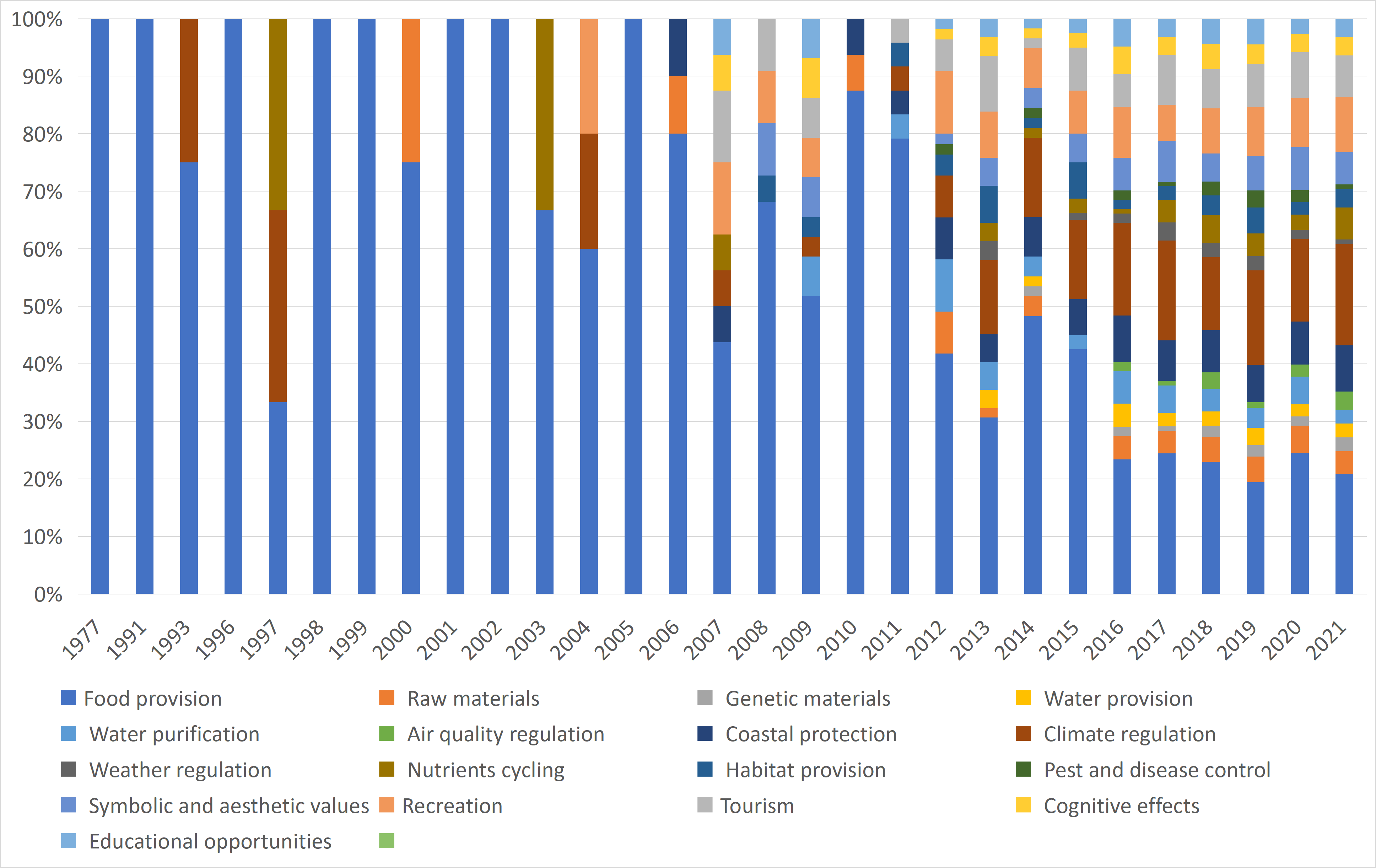


Figure 5 of AF7: Proportion over time of published articles on ES components


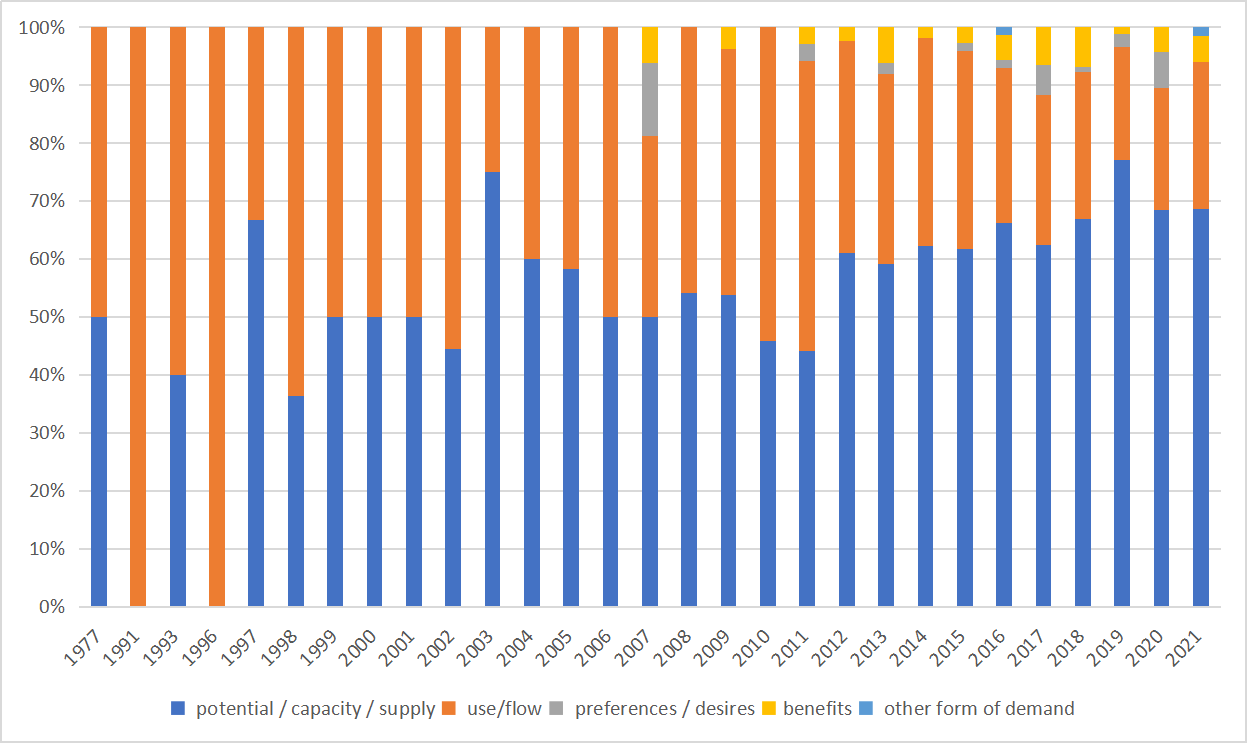


Figure 6 of AF7: Proportion over time of published articles on ES values


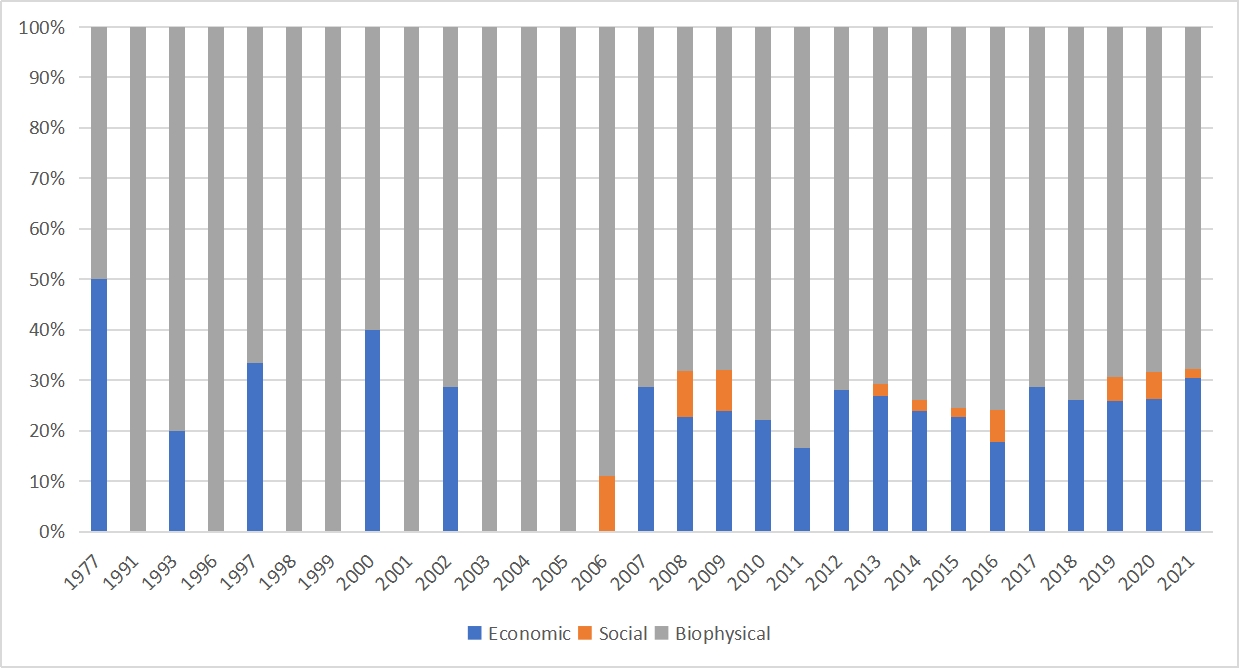


Table 1 of AF7 Distribution of the number of articles per Ecosystem Service and type of data, study design, time frame and time data (the colour of the cells is set according to the high and low values of each column separately)

|  |  |  | Food provision | Raw materials | Genetic materials | Water provision | Water purification | Air quality regulation | Coastal protection | Climate regulation | Weather regulation | Nutrients cycling | Habitat provision | Pest and disease control | Symbolic and aesthetic values | Recreation | Tourism | Cognitive effects | Educational opportunities |
| --- | --- | --- | --- | --- | --- | --- | --- | --- | --- | --- | --- | --- | --- | --- | --- | --- | --- | --- | --- |
|  |  | Total | 433 | 50 | 17 | 28 | 53 | 18 | 89 | 183 | 20 | 41 | 40 | 19 | 64 | 93 | 87 | 42 | 43 |
| Data type | Primary data | 375 | 213 | 34 | 9 | 17 | 31 | 13 | 53 | 135 | 14 | 28 | 24 | 12 | 46 | 61 | 60 | 31 | 32 |
|  | Quantitative data | 645 | 427 | 46 | 15 | 25 | 51 | 18 | 84 | 182 | 19 | 40 | 38 | 18 | 61 | 88 | 84 | 39 | 41 |
|  | Qualitative data | 27 | 19 | 6 | 3 | 4 | 6 | 0 | 10 | 5 | 3 | 2 | 4 | 3 | 10 | 13 | 11 | 8 | 7 |
|  | Data variability | 379 | 230 | 16 | 4 | 7 | 17 | 5 | 34 | 120 | 5 | 20 | 16 | 6 | 24 | 35 | 36 | 18 | 18 |
| Study design | Control-impact design | 21 | 5 | 2 | 1 | 1 | 1 | 0 | 6 | 13 | 1 | 1 | 1 | 1 | 3 | 4 | 4 | 2 | 2 |
|  | Before-after design | 24 | 18 | 4 | 1 | 4 | 6 | 1 | 8 | 3 | 1 | 2 | 5 | 2 | 6 | 6 | 6 | 3 | 3 |
|  | Before-after control-impact design | 5 | 2 | 1 | 0 | 1 | 1 | 0 | 0 | 2 | 0 | 0 | 0 | 0 | 0 | 0 | 0 | 0 | 0 |
|  | Multiple before-after control-impact design | 7 | 3 | 0 | 0 | 0 | 1 | 0 | 2 | 3 | 1 | 2 | 1 | 0 | 1 | 1 | 1 | 1 | 1 |
|  | Multiple impact design | 157 | 65 | 12 | 6 | 7 | 15 | 6 | 27 | 72 | 6 | 19 | 14 | 10 | 26 | 35 | 31 | 16 | 15 |
|  | Multiple impact design an temporal series | 231 | 158 | 19 | 7 | 9 | 19 | 8 | 34 | 61 | 6 | 10 | 9 | 6 | 21 | 27 | 28 | 16 | 16 |
|  | Temporal series along the disturbance | 212 | 186 | 14 | 3 | 8 | 11 | 4 | 17 | 29 | 6 | 7 | 11 | 2 | 11 | 24 | 21 | 6 | 9 |
|  | Temporal series post-disturbance | 8 | 3 | 0 | 0 | 0 | 0 | 0 | 0 | 3 | 0 | 1 | 0 | 0 | 0 | 1 | 0 | 0 | 0 |
|  | Correlation analysis | 160 | 99 | 12 | 3 | 4 | 13 | 3 | 18 | 51 | 4 | 8 | 8 | 4 | 17 | 21 | 22 | 14 | 13 |
| Time frame | Past | 490 | 367 | 42 | 13 | 21 | 37 | 15 | 52 | 112 | 16 | 25 | 30 | 11 | 43 | 63 | 60 | 30 | 31 |
|  | Present | 446 | 278 | 30 | 13 | 21 | 35 | 10 | 67 | 134 | 15 | 27 | 25 | 14 | 42 | 60 | 57 | 26 | 26 |
|  | Future | 146 | 112 | 9 | 2 | 5 | 8 | 2 | 27 | 21 | 2 | 7 | 8 | 2 | 13 | 20 | 17 | 3 | 4 |
| Time data | Observation | 598 | 400 | 48 | 17 | 28 | 51 | 18 | 83 | 166 | 20 | 38 | 38 | 19 | 62 | 90 | 84 | 41 | 42 |
|  | Experimentation | 153 | 42 | 6 | 2 | 5 | 10 | 3 | 15 | 97 | 3 | 18 | 6 | 5 | 15 | 20 | 19 | 12 | 11 |
|  | Projection / prediction | 146 | 112 | 9 | 2 | 5 | 8 | 2 | 27 | 21 | 2 | 7 | 8 | 2 | 13 | 20 | 17 | 3 | 4 |
